# Homeobox D9 drives the malignant phenotypes and enhances the Programmed death ligand-1 expression in non-small cell lung cancer cells via binding to Angiopoietin-2 promoter

**DOI:** 10.1186/s12957-023-02969-z

**Published:** 2023-03-13

**Authors:** Jiabei He, Mengjia Jiang, Jing Liu, Ruiping Zhu, Weipeng Lv, Ruiqing Lian, Yang Yang, Ruoyu Wang

**Affiliations:** 1 The Fourth Department of Oncology, Affiliated Zhongshan Hospital of Dalian University, 6 Jiefang Street, Zhongshan District, Dalian, 116001 Liaoning China; 2 Department of Pathology, Affiliated Zhongshan Hospital of Dalian University, Dalian, 116001 Liaoning China

**Keywords:** Homeobox D9, Angiopoietin-2, Non-small cell lung cancer, Malignant behavior, Programmed death ligand-1

## Abstract

Non-small cell lung cancer (NSCLC) is the leading cause of cancer-related death worldwide. Homeobox D9 (HOXD9), a member of the HOX family of transcription factors, plays a driver role in development of multiple cancers. Angiopoietin-2 (ANGPT2) is reportedly to facilitate angiogenesis, growth and metastasis in various cancers, including lung cancer. In addition, blocking ANGPT2 can effectively improve cancer immunotherapy via downregulation of Programmed death ligand-1 (PD-L1). The purpose of this study was to elucidate the role of HOXD9 in NSCLC and whether ANGPT2 is required for HOXD9-mediated malignant behaviors of NSCLC cells. By performing a series of in vitro functional experiments, we found that knockdown of HOXD9 induced proliferative inhibition, cell cycle G1 arrest, apoptosis, migratory suppression and invasive repression of NSCLC cells. Reduced PD-L1 expression in NSCLC cells was observed after HOXD9 silencing. Besides, HOXD9 deletion decreased the expression of ANGPT2 in NSCLC cells. In line with this, HOXD9 overexpression led to opposite alteration in NSCLC cells. Mechanistically, ANGPT2 was transcriptionally activated by HOXD9. Forced expression of ANGPT2 significantly regulated HOXD9-mediated malignant phenotypes, and enhanced PD-L1 expression of NSCLC cells. Our results expressing HOXD9 may function as an oncogene in NSCLC via trans-activation of ANGPT2.

**Supplementary Information:**

The online version contains supplementary material available at 10.1186/s12957-023-02969-z.

## Introduction

Lung cancer ranks among the most frequent causes of cancer-related deaths worldwide, histologically divided into small cell lung cancer and non-small cell lung cancer (NSCLC). NSCLC represents about 85% of all lung cancer cases, with an overall 5-year survival rate of < 15% [1, 2]. The current cancer treatments, including surgical resection, chemotherapy, immunotherapy and targeted therapy, have contributed to an improvement in the survival of NSCLC patients [3]. Nonetheless, data from clinical research demonstrate that the long-term survival of NSCLC patients remains unsatisfactory [4]. A majority of NSCLC patients die from advanced or metastatic disease [5]. Thus, it is imperative to identify a novel biomarker to improve the early diagnosis of NSCLC.

Homeobox D cluster (HOXD) genes encode a highly conserved family of transcription factors that are involved in controlling cellular and biological processes including cell cycle, cell proliferation, apoptosis, embryonic development, differentiation, and oncogenesis [6]. HOXD9 is one of the HOXD gene located at 2q31 chromosome region. This gene was initially discovered in the development of the forelimb and axial skeleton [7]. Accumulating evidence has suggested the contribution of HOXD9 in initiation and evolution of tumor. Clinically, elevated levels of HOXD9 have been detected in various cancers, such as glioblastomas [8], cervical cancer [9], colorectal carcinoma [10], gastric cancer [11], hepatocellular carcinoma [12], and esophageal squamous cell carcinomas [13]. Deletion of HOXD9 could inhibit tumor cell proliferation and cause cell cycle G1 arrest, as well as weaken tumor-forming capacity [10–12, 14]. Moreover, HOXD9 silencing induced apoptosis of tumor cells by activating the p53 pathway [9]. Recently, HOXD9 was found to be highly expressed in tumor samples from NSCLC patients and had a significant negative correlation with patients’ overall survival [15]. Therefore, HOXD9 is proposed to be a valuable prognostic biomarker for NSCLC. However, the function of HOXD9 in the development and progression of NSCLC remains unknown. We therefore investigated whether HOXD9 serves a central role in endowing malignant behaviors of NSCLC cells.

The formation of new blood vessels (angiogenesis) is required for the growth and metastasis of tumors [16]. Angiopoietin-2 (ANGPT2) as a key factor of angiogenesis drives tumor progression [17]. The published data indicated that enhanced expression or high plasma level of ANGPT2 in NSCLC was associated with patients’ poor prognosis [18, 19]. Furthermore, the oncogenic roles of ANGPT2 were determined in NSCLC [20]. ANGPT2 silencing efficiently inhibited the NSCLC cell A549 proliferation and other malignant behaviors [21]. Besides, a previous study indicated that ANGPT2 played an additional role in immune checkpoint therapy via induction of PD-L1 expression in M2-polarized macrophages [22]. By using JASPAR and PROMO online databases, we found some potential HOXD9-binding sites in the promoter regions of ANGPT2. Therefore, it was hypothesized that HOXD9 might drive malignant phenotypes and induce PD-L1 expression in NSCLC cells by regulating ANGPT2.

In the current study, both gain- and loss-of-function experiments of HOXD9 were performed to assess its role in the proliferation, cell cycle and apoptosis of NSCLC cells. Furthermore, the effect of HOXD9 on PD-L1 expression in NSCLC cells was investigated.

## Materials and methods

### Acquisition of gene expression omnibus (GEO) data

The expression data of GSE118370 for HOXD9 were obtained from the GEO database (http://www.ncbi.nlm.nih.gov/geo/) to compare the expression of HOXD9 between NSCLC tissues and paired normal lung tissues.

### Tissue samples

A total of 30 cases of lung cancer tissues and their corresponding paracancerous tissues were collected from Affiliated Zhongshan Hospital of Dalian University from August 2021 to August 2022. No patients received radiotherapy or chemotherapy before surgery. The study protocol was approved by the medical ethics committee of Affiliated Zhongshan Hospital of Dalian University. All tissue samples were obtained with informed consent from patients.

### Cell culture

Human NSCLC cell lines NCI-H661 and NCI-H292, obtained from iCell Bioscience Inc. (Shanghai, China), were cultured in RPMI 1640 (Cat No., 31,800, Solarbio, Beijing, China) supplemented with 10% fetal bovine serum (FBS; Cat No., 11,011–8611, Solelybio, Hangzhou, China). HEK293T cells were purchased from ZQXZ Biotechnology Co., Ltd., Shanghai, China and incubated in dulbecco’s modified eagle medium (DMEM; Cat No., G4510, Servicebio, Wuhan, China) containing 10% FBS. All cells were maintained in an incubator at 37 °C with 5% CO_2_.

### Cell transfection

For HOXD9 knockdown, two small interfering RNAs targeting HOXD9 (siHOXD9^#1^ sense: 5’-AGAAAGAAUUCCUCUUCAA-3’, anti-sense: 5’-UUGAAGAGGAAUUCUUUCU-3’; siHOXD9^#2^ sense: 5’-GCUUGAGCUGGAGAAAGAA-3’, anti-sense: 5’-UUCUUUCUCCAGCUCAAGC-3’) and their negative control (siNC sense: 5’-UUCUCCGAACGUGUCACGU-3’, anti-sense: 5’-ACGUGACACGUUCGGAGAA-3’) were obtained from GenePharma Co., Ltd., Shanghai, China. Cell transfection was performed after adherence. NCI-H292 cells were transfected with siHOXD9 or siNC using Lipofectamine 3000 (Cat No., L3000 008, Invitrogen, Carlsbad, CA, USA) following the manufacturer’s protocol. For HOXD9 overexpression, the HOXD9 overexpressing plasmid (exHOXD9) and empty vector were obtained from ZQXZ Biotechnology. NCI-H661 or HEK293T cells were transfected with exHOXD9 or vector using Lipofectamine 3000. To determine whether ANGPT2 was involved in HOXD9-mediated pathological mechanisms, the plasmid expressing ANGPT2 (exANGPT2; ZQXZ Biotechnology) and siHOXD9 were co-transfected into NCI-H292 cells using Lipofectamine 3000. The exHOXD9 and a small interfering RNA targeting ANGPT2 (siANGPT2 sense: 5’-GGAUGGAGACAACGACAAA-3’; anti-sense: 5’-UUUGUCGUUGUCUCCAUCC-3’) or its negative control (siNC sense: 5’-UUCUCCGAACGUGUCACGU-3’; anti-sense: 5’-ACGUGACACGUUCGGAGAA-3’) were co-transfected into NCI-H661 cells using Lipofectamine 3000.

### Cell counting kit-8 (CCK-8) assay

Cells were seeded in a 96-well microplate at a density of 1 × 10^4^ cells/well. After transfection, cells were incubated at 37 °C with 5% CO_2_ for 0 h, 24 h, 48 h and 72 h, respectively. Each well was added to 10µL of CCK-8 reagent (Cat No., KGA317, KeyGene Biotech., Nanjing, China) to be cultured for 2 h again. Finally, optical density (OD) value at 450 nm was measured using a microplate reader to determine cell viability.

### 5-ethynyl-2’-deoxyuridine (EdU) staining

Cell proliferation assay was performed using a kFluor647 Click-iT EdU Imaging Test Kit (Cat No., KGA335, KeyGene Biotech.). Briefly, cells were pulsed with 10 μM EdU solution and incubated at 37 °C with 5% CO_2_ for 2 h. After fixing with 4% paraformaldehyde for 15 min, cells were permeabilized with 0.5% Triton X-100 for 20 min, incubated with Click-iT reaction mixture in the dark for 30 min and counterstained with 4’, 6’-diamidino-2-phenylindole (DAPI) for 5 min, at room temperature. The images of the cells were photographed using a fluorescence microscope at 400 × magnification. Cell proliferation rate was calculated as the following formulation: (EdU-positive cells/total cells) × 100%. The number of EdU-positive cells was counted manually in three random microscopic fields.

### Correlation analysis

UALCAN (http://ualcan.path.uab.edu/index.html) was a comprehensive web resource, which provided RNA-seq and clinical data of various cancer types based on the cancer genome atlas (TCGA) [23]. TIMER2.0 (http://timer.cistrome.org/) is a reliable and comprehensive resource that allows exploring tumor immunological, clinical and genomic features [24]. In this study, UALCAN and TIMER2.0 were applied to analyze the correlation between genes in NSCLC.

### Dual-luciferase assay

HEK293T cells were collected at 90% confluency. Cells were co-transfected with a pGL3-basic vector containing the promoter sequence of *homo* ANGPT2, pRL-TK-Renilla luciferase reporter vector and exHOXD9. After 24 h of transfection, cells were lysed and lysed samples were analyzed for firefly and renilla luciferase activities by a Dual Luciferase Reporter Gene Assay Kit (Cat No., KGAF040, KeyGene Biotech.). Relative luciferase activity was calculated by normalizing firefly to renilla luciferase activity.

### Enzyme-linked immunosorbent assay (ELISA)

A human PD-L1 ELISA Kit [Cat No., EK1261, Multisciences (Lianke) BioTECH., Hangzhou, China] was used to detect the soluble PD-L1 level. An antibody specific for PD-L1 were pre-coated onto the microwells. After centrifugation, cell supernatants were added to microwells and incubated with streptavidin-HRP, followed by the coloration of tetramethyl-benzidine reagent. Finally, the OD value at 450 nm was measured using a microplate reader.

### Real-time polymerase chain reaction (PCR)

Total RNA extraction from cells was performed using a TRIpure Kit (Cat No., RP1001, Bioteke, Beijing, China). Complementary DNA (cDNA) was synthesized from extracted total RNA using BeyoRT II M-MLV reverse transcriptase (Cat No., D7160L, Beyotime, Shanghai, China). Afterwards, real time-PCR assay was performed using cDNA template, primers, SYBR Green (Cat No., SY1020, Solarbio), and 2 × Taq PCR MasterMix (Cat No., PC1150, Solarbio). Primers used for real time-PCR were obtained from Genscript (Piscataway, NJ, USA) and their sequences were listed as follows: *HOXD9*, 5-TCCTCCACTTCCTTATCCTCC-3 (forward), 5-TCCTCCTTCAGCGAACAGC-3 (reverse); *ANGPT2*, 5-GGATTTGGTAACCCTTCA-3 (forward), 5-ATTCCCTTCCCAGTCTTT-3 (reverse); *GAPDH*, 5-GACCTGACCTGCCGTCTAG-3 (forward), 5-AGGAGTGGGTGTCGCTGT-3 (reverse). Relative expression of gene was determined by the delta-delta Ct method [25]. GAPDH was used as a housekeeping gene to normalize expression levels of genes.

### Scratch-wound healing migration assay

Scratch-wound healing experiment was performed to assess migratory ability of NSCLC cells. A scratch was made by using a 200 μl-pipette tip on the cell monolayer in the presence of 1 μg/ml mitomycin C. The wound healing was monitored at 0 h and 24 h post-scratching under an inverted phase contrast microscope (magnification × 100). The migratory rate was calculated as the ratio of wound width at 24 h and wound width at 0 h.

### Transwell matrigel invasion assay

Cell invasive ability was evaluated using a Matrigel-coated Transwell membrane filter inserts with 8-μm pores. Cells (5 × 10^4^ in 200 µl of serum-free culture medium) were seeded in the upper surface of membranes, and allowed to invade towards the bottoms of 24-well plates containing 800 μl complete growth medium with 10% FBS for 24 h. After wiping the non-invaded cells with sterile cotton buds, the invaded cells were fixed with 4% paraformaldehyde for 20 min and then stained with 0.5% crystal violet for 5 min. The invaded cells were counted under an inverted microscope (magnification × 200) in five random fields.

### Western blot

Total protein from cells was isolated in radioimmunoprecipitation (RIPA) lysis buffer (Cat No., R0010, Solarbio) supplemented with 1 mM phenylmethanesulfonyl fluoride (PMSF; Cat No., P0100, Solarbio). The Bicinchoninic Acid (BCA) Protein Assay Kit (Cat No., PC0020, Solarbio) was used to measure the protein concentration of lysates. Protein was separated by sodium dodecyl sulfate polyacrylamide gel electrophoresis (8%, 10%, 15% gel), followed by electrotransfer of proteins from the gel to a PVDF membrane (Cat No., IPVH00010, Millipore, Billerica, MA, USA). After blocking with 5% (m/v) skim milk (Cat No., A600669, Sangon Biotech, Shanghai, China), the membrane was probed with primary antibodies against HOXD9 (1:1000 dilution, rabbit polyclonal; Cat No., 20,560–1-AP, ProteinTech, Wuhan, China), cleaved poly (ADP-ribose) polymerase 1 (PARP1; 1:500 dilution, rabbit monoclonal; Cat No., 5625, CST, Danvers, MA, USA), cleaved caspase-3 (1:1000 dilution, rabbit monoclonal; Cat No., 9661, CST), cyclin E (1:500 dilution, rabbit polyclonal; Cat No., AF0144, Affinity Biosciences, Changzhou, China), cyclin B1 (1:1000 dilution, rabbit monoclonal; Cat No., A19037, ABclonal, Wuhan, China), ANGPT2 (1:1000 dilution, rabbit monoclonal; Cat No., A11306, ABclonal), PD-L1 (1:1000 dilution, rabbit polyclonal; Cat No., DF6526, Affinity Biosciences) and GAPDH (1:10,000 dilution, mouse monoclonal; Cat No., 60,004–1-Ig, ProteinTech) at 4 °C overnight. Subsequently, the membrane was incubated with the correspondingly horseradish peroxidase (HRP)-conjugated secondary antibodies (1:3000 dilution; Cat No., SE134/SE131, Solarbio) at 37 °C for 1 h. Protein bands on the membrane were visualized by enhanced chemiluminescence (Cat No., PE0010, Solarbio). GAPDH was used as an internal control.

### Flow cytometry

Cell cycle detection was performed using a Cell Cycle Analysis Kit (Cat No., C1052, Beyotime). Cells (4 × 10^5^ cells/well) were seeded in a 6-well plate. After transfection, cells were fixed in ethanol (70%) at 4 °C for 12 h, followed by centrifugation and resuspension in staining buffer. Cells were then incubated with 25 μL propidium iodide (PI) and 10 μL RNase A in the dark at 37 °C for 30 min. Cell cycle was detected using a NovoCyte flow cytometer. The proportion of cells in each stage of the cell cycle was quantified by NovoExpress software. Cell apoptosis was detected using a commercial kit (Cat No., KGA106, KeyGene Biotech.). Briefly, cells were centrifuged and resuspended in 500 μL binding buffer. Afterwards, cells were double-stained with 5 μL Annexin V-FITC and 5 μL PI. After incubating in the dark at 37 °C for 30 min, cell apoptosis was detected using the NovoCyte flow cytometer. Apoptosis was analyzed using NovoExpress by dividing the cells into four quadrants of dot plots as: necrotic cells (Annexin V^−^/PI^+^ appearing in Q1 quadrant), late apoptotic cells (Annexin V^+^/PI^+^ in Q2 quadrant), viable cells (Annexin V^−^/PI^−^ in Q3 quadrant), and early apoptotic cells (Annexin V^+^/PI^−^ in Q4 quadrant). The apoptotic rate was calculated by adding the percentages of cells presented in Q2 and Q4 quadrants. For cell surface PD-L1 on lung cancer cell lines, cells centrifuged and resuspended in 100 μL phosphate buffer saline. Cells were then incubated with 0.5 μL FITC anti-human CD274 antibody (Cat No., 374,509, Biolegend, San Diego, CA, USA) in the dark at 37 °C for 30 min. After centrifugation, cells were resuspended in 100 μL cell stain buffer and measured analyzed using flow cytometer.

### Statistical analysis

All data were analyzed using GraphPad Prism 8.0 software and expressed as mean ± SD. Statistical significance among two groups was determined using Student’s t-test. One-way analysis of variance post hoc Tukey’s test was used for multi-group comparison. A *p* < 0.05 was regarded as statistical significance.

## Results

### HOXD9 is an upregulated gene that predicts worse overall survival in patients with NSCLC

**Fig. 1:**
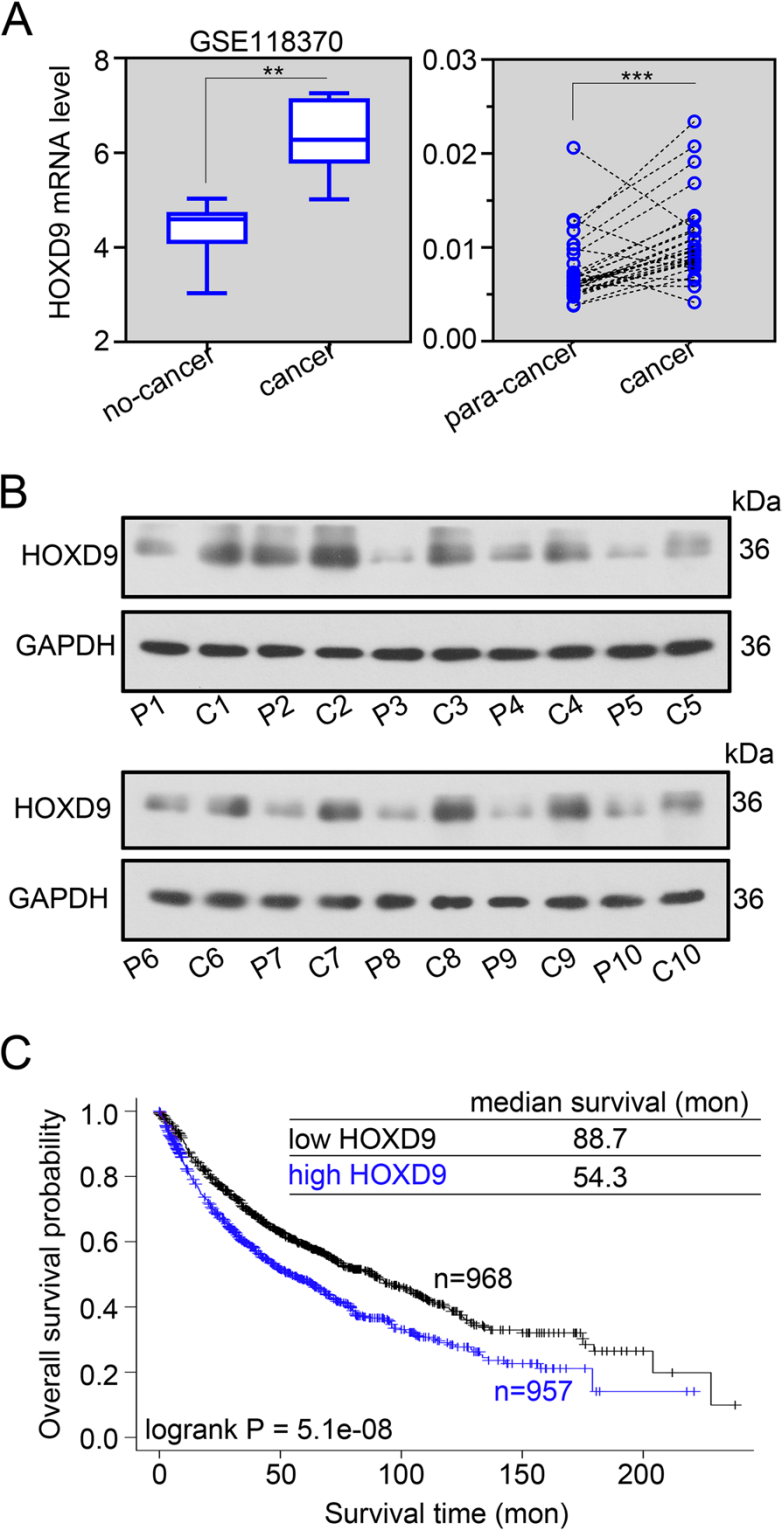
HOXD9 is an upregulated gene that predicts worse overall survival in patients with NSCLC. **A** HOXD9 mRNA levels in NSCLC and normal lung tissues obtained from GSE118370 were detected (left). HOXD9 mRNA levels in 30 paired NSCLC and para-NSCLC tissues from our independent hospital cohorts were detected via real time-PCR (right). **B** HOXD9 protein levels in 10 paired NSCLC and para-NSCLC tissues from our independent hospital cohorts were detected via western blot. P, para-NSCLC tissue; C, NSCLC tissue. **C** The effect of HOXD9 on the overall survival in NSCLC patients via Kaplan–Meier Plotter. Patients with NSCLC expressing high HOXD9 levels are labeled in blue, whereas those with tumors with low HOXD9 levels are shown in black. ** *p* < 0.01; *** *p* < 0.001. Data in (**A**) are presented as mean values ± SD. The blots were cropped and the original uncropped images of blots were shown in supplementary materials

We analyzed the expression levels of HOXD9 in NSCLC tissues using the GEO and our independent hospital databases. Results revealed that the mRNA levels of HOXD9 were significantly increased in NSCLC samples when compared with non-NSCLC tissues (normal lung tissues and para-NSCLC tissues) from GSE1183370 (Fig. 1A, left) and independent hospital cohorts (Fig. 1A, right). We also examined HOXD9 protein expression in NSCLC and para-NSCLC tissues from independent hospital cohorts via western blot, which showed that HOXD9 protein expression was remarkably increased in cancer tissues (Fig. 1B). The effect of HOXD9 expression on the overall survival of patients with NSCLC was analyzed via Kaplan–Meier Plotter (www.kmplot.com). Results suggested the worse overall survival in NSCLC patients with high HOXD9 expression (Fig. 1C).

### HOXD9 drives the malignant biological behaviors in NSCLC cells

**Fig. 2:**
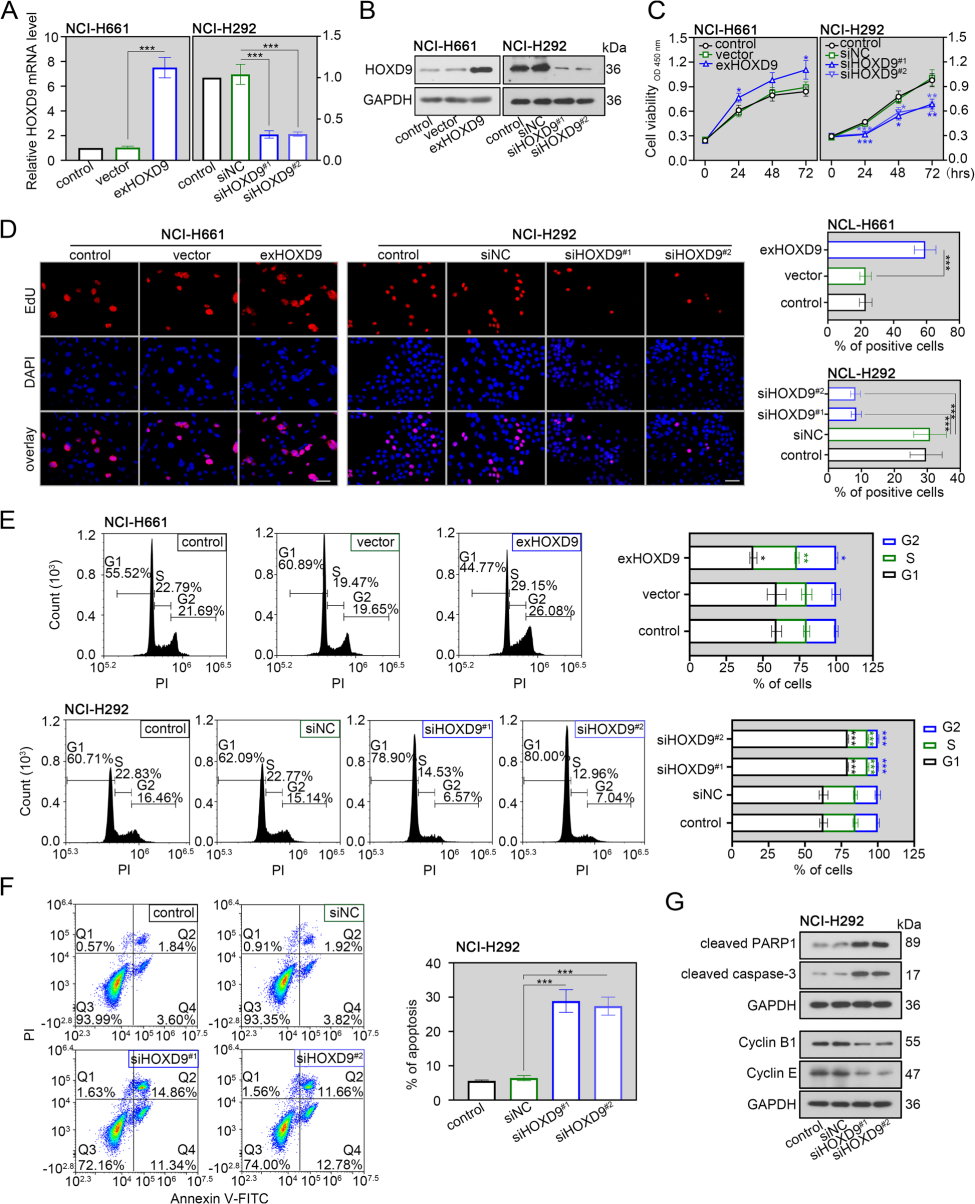
HOXD9 induces proliferation, cell cycle progress and apoptotic inhibition of NSCLC cells. NCI-H661 cells were transfected with HOXD9 overexpressing plasmid (exHOXD9) or empty vector, while NCI-H292 cells were transfected with two small interfering RNAs targeting HOXD9 (siHOXD9^#1^, siHOXD9^#2^) or none-specific sequence (siNC). **A** After 48 h of transfection, HOXD9 mRNA expression in two NSCLC cells (NCI-H661 and NCI-H292) was evaluated by real time-PCR assay. **B** After 48 h of transfection, HOXD9 protein expression in two NSCLC cells was evaluated by western blot assay. **C** After 48 h of transfection, NSCLC cells were seeded into 96-well microplates and analyzed with CCK-8 reagents at indicated time. **D** After 48 h of transfection, NSCLC cells were subjected into the EdU incorporation assay. The new generation cells were stained via EdU (red). DAPI stained nuclei in blue. Scale bar = 50 μm. Quantification of EdU-positive cells was performed to assess cell proliferation. **E** Cell cycle progression in two NSCLC cells was evaluated by flow cytometry analysis. **F** Apoptosis in NCI-H292 cells was evaluated by flow cytometry analysis. The apoptotic rate was quantified by adding the percentages of early apoptotic cells (Annexin V^+^/PI^−^ in Q4 quadrant) and late apoptotic cells (Annexin V^+^/PI^+^ in Q2 quadrant). **G** Expression of protein markers involved in apoptosis and cell cycle regulation in NCI-H292 cells was evaluated by western blot assay. * *p* < 0.05; ** *p* < 0.01; *** *p* < 0.001. Data in (**A**), (**C**), (**D**), (**E**) and (**F**) are presented as mean values ± SD. The blots were cropped and the original uncropped images of blots were shown in supplementary materials

The functional impact of HOXD9 on cell behaviors in vitro was assessed. Based on a database from CCLE (https://sites.broadinstitute.org/ccle), knockdown of HOXD9 was performed in NCI-H292 cells with relatively high HOXD9 expression, while HOXD9 overexpression was conducted in NCI-H661 cells with relatively low HOXD9 expression. The use of overexpression plasmid could effectively upregulated HOXD9 mRNA and protein expression (Fig. 2A and B). Also, two siRNAs were designed to silence HOXD9 expression and reduce off-target effects (Fig. 1A and B). Afterwards, the CCK-8 assay and EdU staining were carried out to evaluate the ability of cell proliferation. The proliferative ability was inhibited in NCI-H292 cells after HOXD9 silencing, whereas highly expressing HOXD9 promoted cell proliferation (Fig. 2C and D). By performing flow cytometry analysis, we found that knocking down HOXD9 induced the population shift to the G1 phase of NCI-H292 cell lines (Fig. 2E). On the contrary, high expression of HOXD9 decreased the population of cells in G1 phase (Fig. 2E). This indicated that silencing of HOXD9 induced cell-cycle arrest in the G1 phase of NSCLC. We also observed a significant increase of apoptotic rate in NCI-H292 cells after HOXD9 silencing (Fig. 2F).

**Fig. 3:**
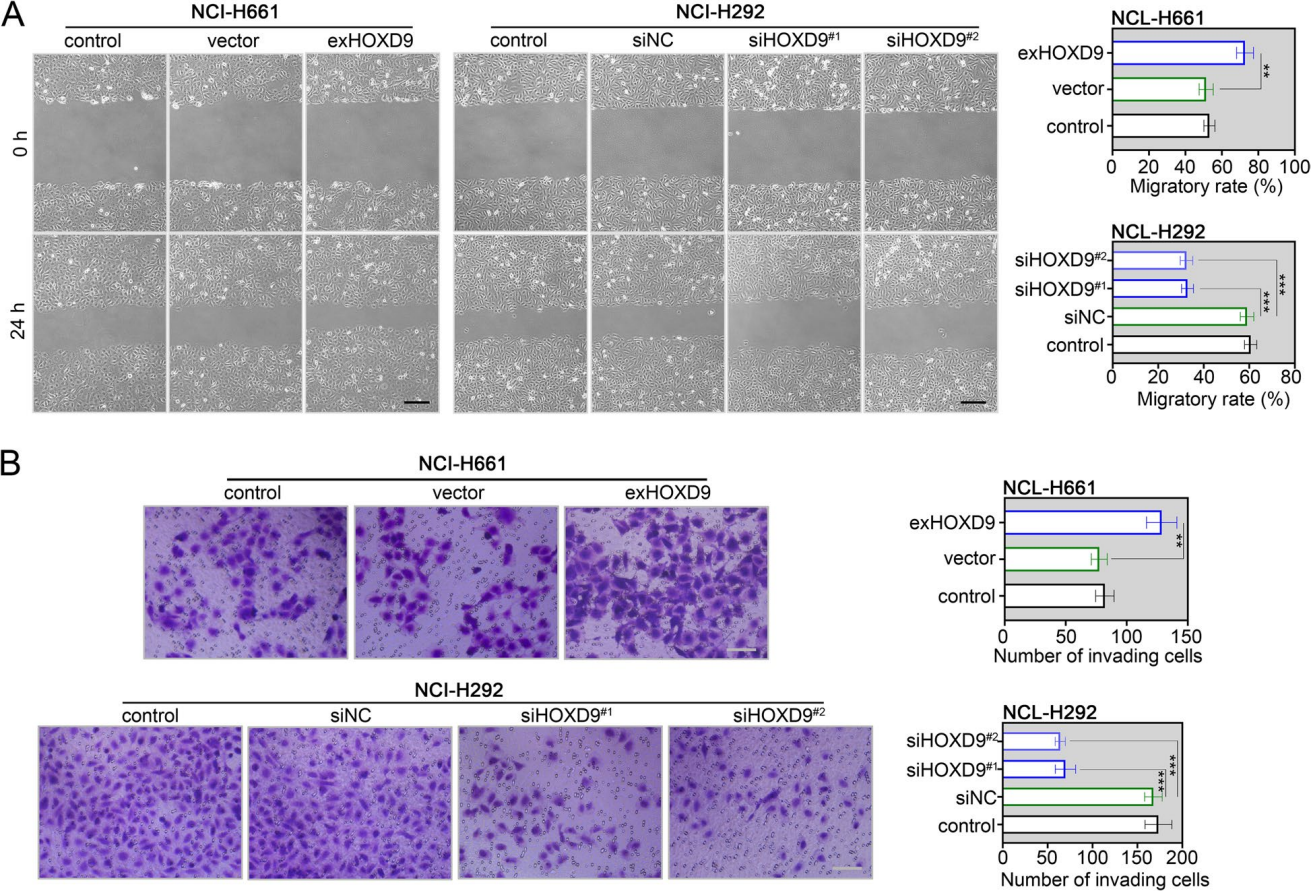
HOXD9 promotes migration and invasion of NSCLC cells. NCI-H661 cells were transfected with exHOXD9 or empty vector, while NCI-H292 cells were transfected with siHOXD9 or siNC. **A** After 48 h of transfection, the migratory ability of two NSCLC cells (NCI-H661 and NCI-H292) was evaluated by scratch-wound healing assay. The wound healing was monitored at 0 h and 24 h post-scratching, and the percentage of wound closure was measured. Scale bar = 200 μm. **B** After 48 h of transfection, the invasiveness of two NSCLC cells was assessed by transwell assay. The invaded cells were counted in five random fields. Scale bar = 100 μm. ** *p* < 0.01; *** *p* < 0.001. Data in (**A**) and (**B**) are presented as mean values ± SD

The effect of HOXD9 on migration and invasion of TSCC cells was further evaluated. By performing the scratch-wound healing experiment, overexpression of HOXD9 accelerated wound closure of NCI-H661 cells (Fig. 3A). In addition, the Transwell invasion assay revealed that highly expressing HOXD9 increased the number of invading cells in NCI-H661 cells (Fig. 3B). In line with this, silencing of HOXD9 dampened the migratory ability and invasiveness of NCI-H292 cells (Fig. 3B). These in vitro data showed that expression of HOXD9 was correlated with the malignant biological behaviors of NSCLC cells.

### ANGPT2 is a direct transcriptional target of HOXD9

**Fig. 4:**
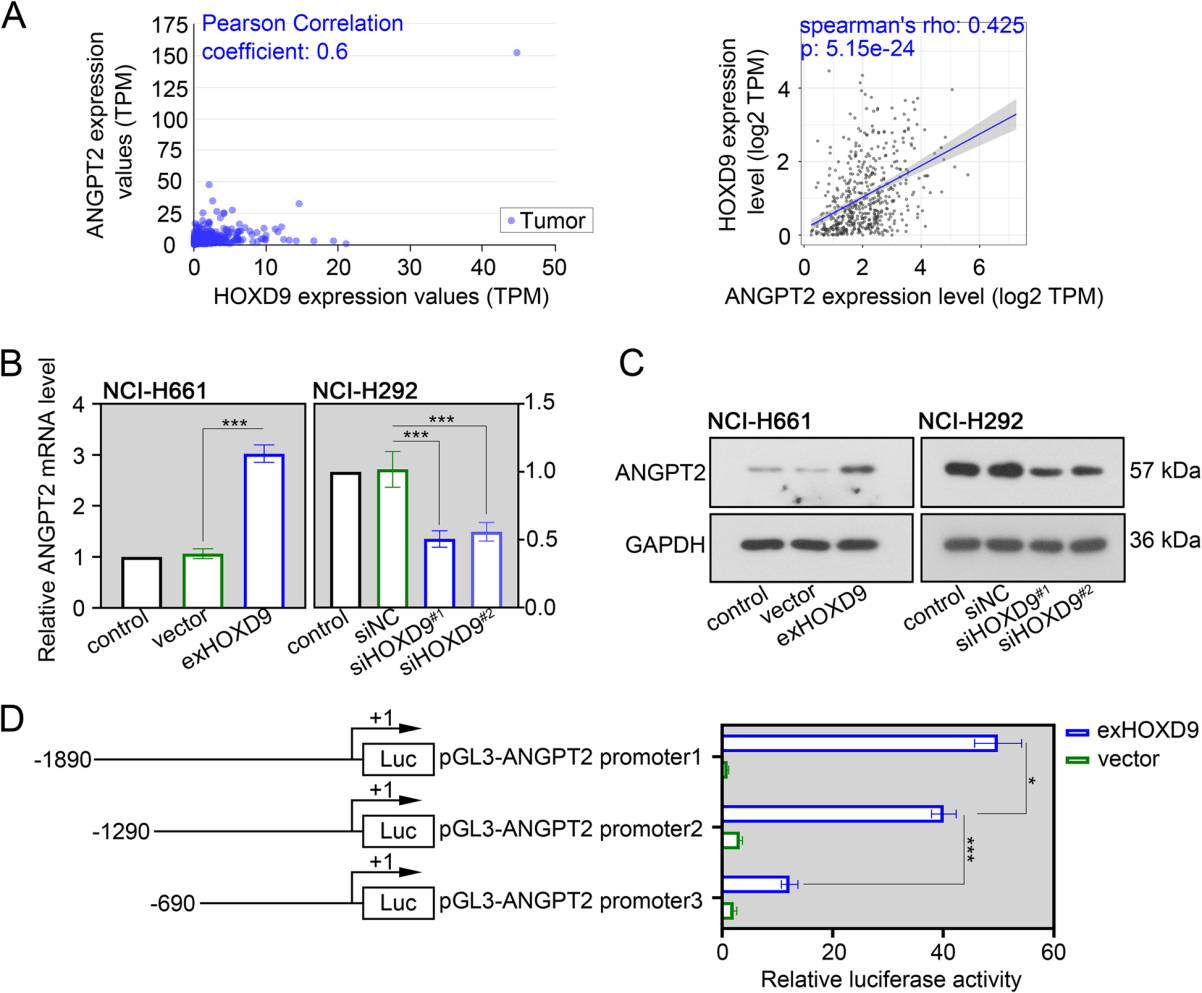
ANGPT2 is a direct transcriptional target of HOXD9. **A** Correlation analysis of HOXD9 with ANGPT2 in NSCLC using the UALCAN and TIMER databases. **B** NCI-H661 cells were transfected with exHOXD9 or empty vector, while NCI-H292 cells were transfected with siHOXD9^#1^, siHOXD9^#2^ or their negative control siNC. After 48 h of transfection, ANGPT2 mRNA expression in two NSCLC cells (NCI-H661 and NCI-H292) was evaluated by real time-PCR assay. **C** After 48 h of transfection, ANGPT2 protein expression in two NSCLC cells was evaluated by western blot assay. **D** HEK293T cells were co-transfected with a pGL3-basic vector containing the promoter sequence of *homo* ANGPT2, pRL-TK, and exHOXD9 or empty vector. After 24 h of transfection, ANGPT2 luciferase activity was evaluated by dual-luciferase reporter assay. * *p* < 0.05; *** *p* < 0.001. Data in (**B**) and (**D**) are presented as mean values ± SD. The blots were cropped and the original uncropped images of blots were shown in supplementary materials

By analyzing data from the UALCAN and TIMER databases, we found that expression of HOXD9 had significant positive correlation with ANGPT2 in NSCLC (Fig. 4A). Additionally, two online databases, JASPAR (https://jaspar.uio.no/) and PROMO (http://alggen.lsi.upc.es), revealed that HOXD9 may bind to the promoter region of ANGPT2. Given the above findings, we detected the ANGPT2 expression by real time-PCR and western blot assays. Results showed that ANGPT2 expression was significantly upregulated after overexpression of HOXD9, yet downregulated after HOXD9 silencing (Fig. 4B and C). Results of dual-luciferase reporter assay confirmed that highly expressing HOXD9 significantly enhanced the relative luciferase activity of ANGPT2 (Fig. 4D), indicating that HOXD9 transcriptionally regulated ANGPT2 expression via binding to ANGPT2 promoter. Moreover, deletion of fragments from -1890 to -1291 bp reduced the luciferase activity by ~ 20%, and deletion of fragments from -1290 to -691 bp reduced the promoter activity by ~ 70%. Such evidence suggested that the HOXD9 may be a strong binding ability with the sites in the promoter region (-1290 ~ -691 bp) of ANGPT2 (Fig. 4D).

### ANGPT2 is required for HOXD9-mediated malignant biological behaviors of NSCLC cells

**Fig. 5:**
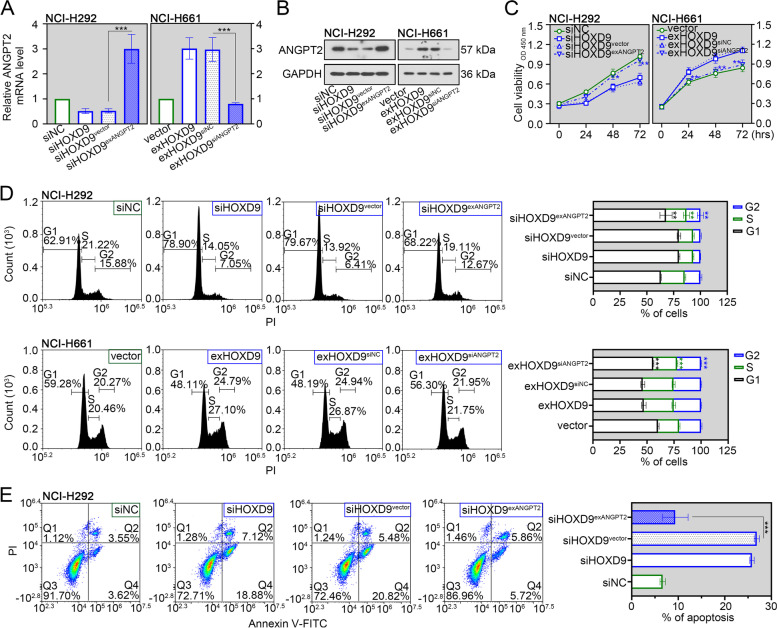
ANGPT2 is required for HOXD9-mediated proliferation, cell cycle progress and apoptotic inhibition of NSCLC cells. NCI-H292 cells were co-transfected with siHOXD9 and the plasmid expressing ANGPT2 (exANGPT2) or empty vector. NCI-H661 cells were co-transfected with exHOXD9 and siANGPT2 or siNC. **A** After 48 h of transfection, ANGPT2 mRNA expression in two NSCLC cells (NCI-H661 and NCI-H292) was evaluated by real time-PCR assay. **B** After 48 h of transfection, ANGPT2 protein expression in two NSCLC cells (NCI-H661 and NCI-H292) was evaluated by western blot assay. **C** After 48 h of transfection, NSCLC cells were seeded into 96-well microplates and analyzed with CCK-8 reagents at indicated time. **D** Cell cycle progression in two NSCLC cells was evaluated by flow cytometry analysis. **E** Apoptosis in NCI-H292 cells was evaluated by flow cytometry analysis. The apoptotic rate was quantified by adding the percentages of early apoptotic cells (Annexin V^+^/PI^−^ in Q4 quadrant) and late apoptotic cells (Annexin V^+^/PI^+^ in Q2 quadrant). * *p* < 0.05; ** *p* < 0.01; *** *p* < 0.001. Data in (**A**), (**C**), (**D**) and (**E**) are presented as mean values ± SD. The blots were cropped and the original uncropped images of blots were shown in supplementary materials

**Fig. 6:**
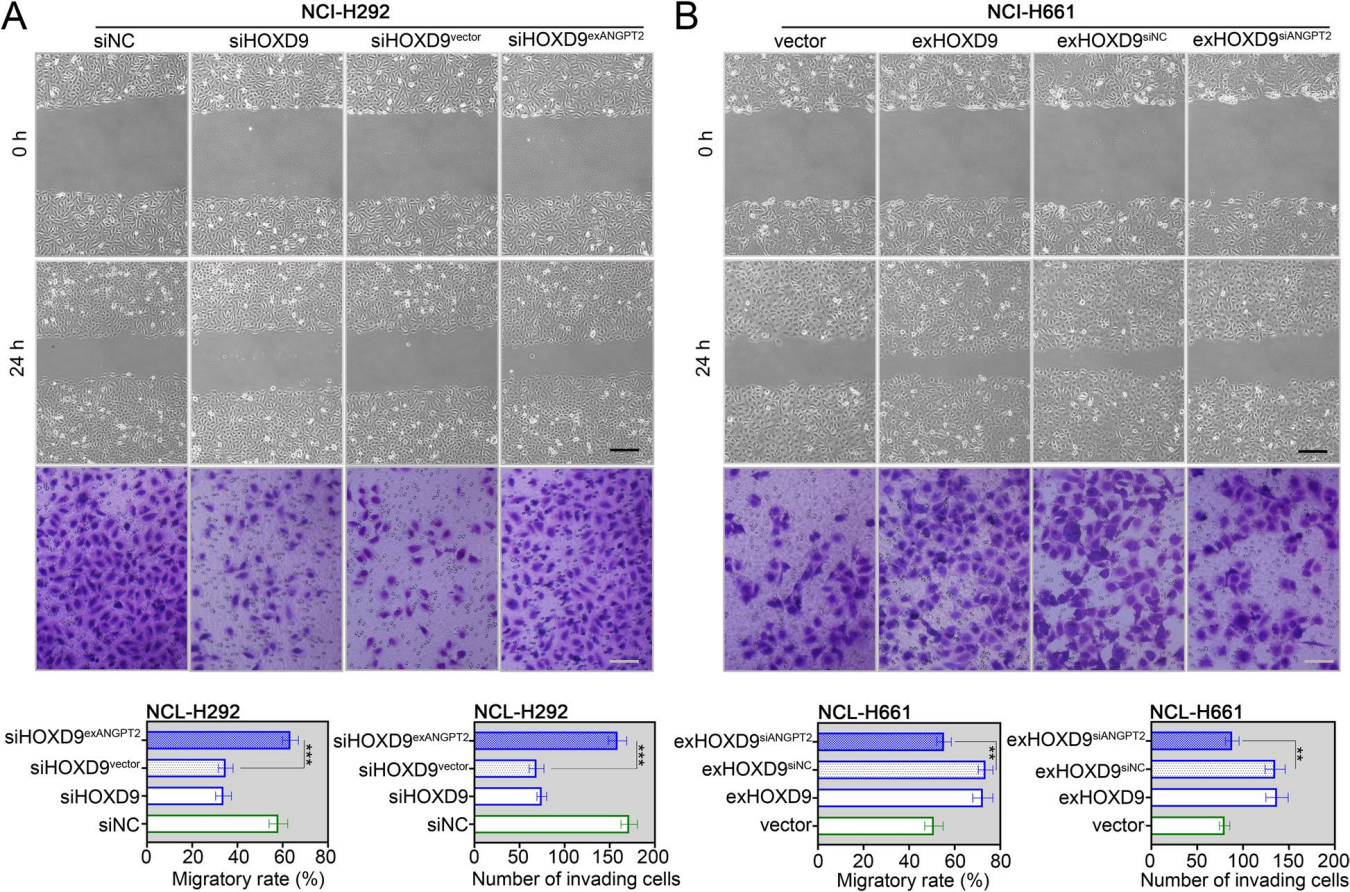
ANGPT2 is involved in the anti-migratory and anti-invasive effects of HOXD9 silencing in NSCLC cells. NCI-H292 cells were co-transfected with siHOXD9 and exANGPT2 or empty vector. NCI-H661 cells were co-transfected with exHOXD9 and siANGPT2 or siNC. (A and B) After 48 h of transfection, the migratory ability and invasiveness of NCI-H292 (**A**) and NCI-H661 (**B**) cells were evaluated by scratch-wound healing assay (Scale bar = 200 μm) and transwell assay (Scale bar = 100 μm). ** *p* < 0.01; *** *p* < 0.001. Data in (**A**) and (**B**) are presented as mean values ± SD

Subsequent experiments were performed to validate whether ANGPT2 was involved in HOXD9-mediated malignant behaviors of NSCLC cells. ANGPT2 expression was examined via real time-PCR and western blot analyses. Results revealed that the levels of ANGPT2 mRNA and protein were increased after co-transfection with siHOXD9 and exANGPT2 into NCI-H292 cells, while the ANGPT2 levels were decreased in NCI-H661 cells co-transfection with exHOXD9 and siANGPT2 (Fig. 5A and B). Following functional experiments suggested that overexpression of ANGPT2 weakened HOXD9 silencing-induced proliferative inhibition, cell cycle arrest, apoptosis, migratory suppression and invasive repression in NCI-H292 cells (Figs. 5C-E and 6A, B). In line with this, ANGPT2 knockdown blocked the HOXD9 overexpression-mediated malignant biological behaviors of NCI-H661 cells (Figs. 5C, D and 6A, B).

### HOXD9 has the ability to stimulate PD-L1 expression in NSCLC cells

**Fig. 7:**
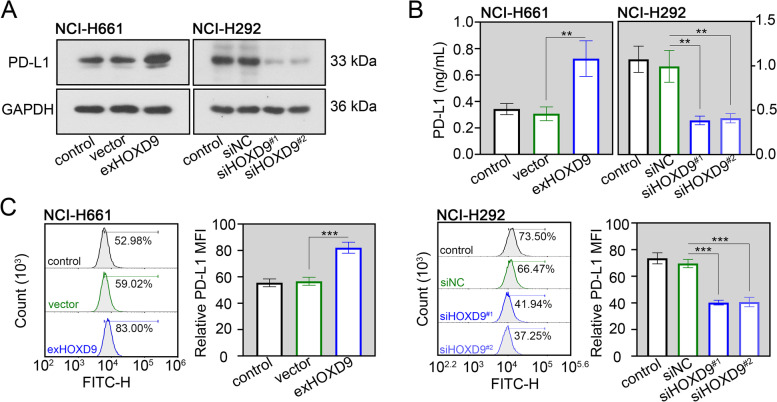
HOXD9 has the ability to stimulate PD-L1 expression in NSCLC cells. NCI-H661 cells were transfected with exHOXD9 or empty vector, while NCI-H292 cells were transfected with siHOXD9^#1^, siHOXD9^#2^ or their negative control siNC. **A** After 48 h of transfection, total PD-L1 protein expression in two NSCLC cells (NCI-H661 and NCI-H292) was evaluated by western blot assay. **B** After 48 h of transfection, soluble PD-L1 protein expression in two NSCLC cells was evaluated by ELISA assay. **C** After 48 h of transfection, membrane PD-L1 protein expression in NCI-H292 cells was evaluated by flow cytometry analysis. MFI, relative mean fluorescence intensity. ** *p* < 0.01; *** *p* < 0.001. Data in (**B**) and (**C**) are presented as mean values ± SD. The blots were cropped and the original uncropped images of blots were shown in supplementary materials

PD-L1 on the cell membrane helps tumor cells to evade T cell-mediated immune surveillance via binding to PD-1 on T cells [26]. High expression of PD-L1 in NSCLC is implicated in poor clinical outcomes [27]. Firstly, we evaluated the effect of HOXD9 on PD-L1 expression in NSCLC cells. Results of western blot assay suggested that the PD-L1 protein level was significantly increased in two NSCLC cell lines after HOXD9 overexpression, yet decreased after HOXD9 silencing (Fig. 7A). As an important immune checkpoint molecule, PD-L1 has a soluble form and a membrane-bound form [28]. By performing the ELISA and flow cytometry analyses, we found that the overexpression of HOXD9 not only increased membrane PD-L1 level, but also induced the release of soluble PD-L1 from NCI-H292 and NCI-H661 cells (Fig. 7B and C). Knockdown of HOXD9 resulted in the opposite alteration (Fig. 7B and C).

### ANGPT2 blocks the suppression of PD-L1 expression by HOXD9 in NSCLC cells

**Fig. 8:**
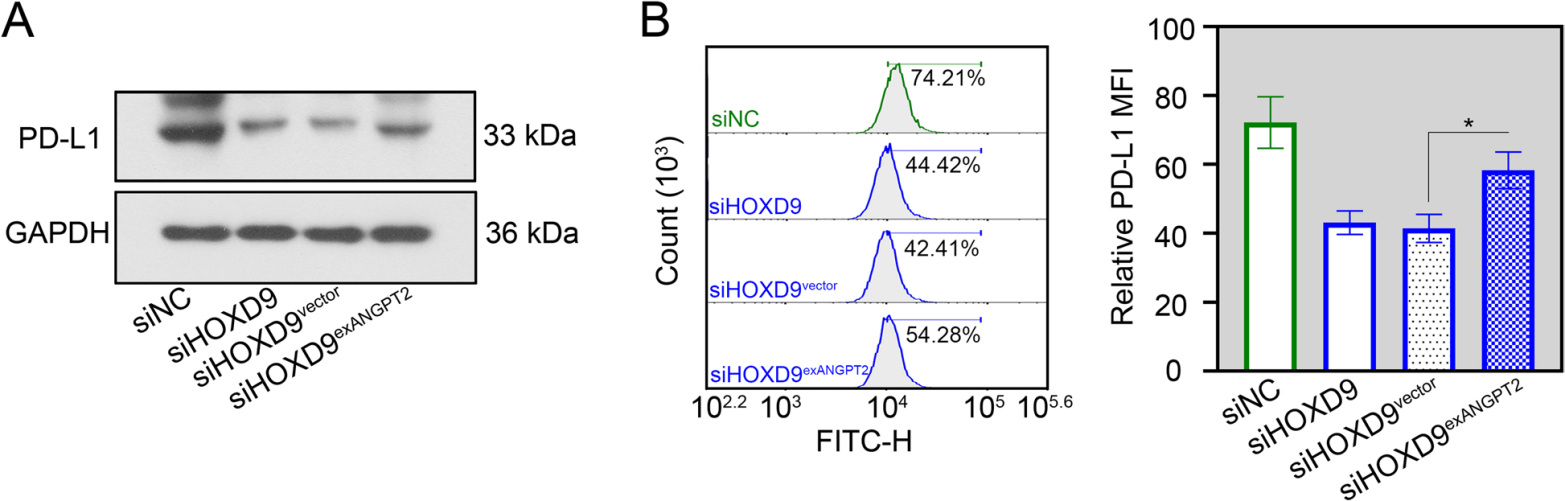
ANGPT2 blocks the suppression of PD-L1 expression by HOXD9 in NSCLC cells. NCI-H292 cells were co-transfected with siHOXD9 and exANGPT2 or empty vector. **A** After 48 h of transfection, total PD-L1 protein expression in NCI-H292 cells was evaluated by western blot assay. **B** After 48 h of transfection, membrane PD-L1 protein expression in NCI-H292 cells was evaluated by flow cytometry analysis. MFI, relative mean fluorescence intensity. * *p* < 0.05. Data in (**B**) are presented as mean values ± SD. The blots were cropped and the original uncropped images of blots were shown in supplementary materials

With the aim to assess whether ANGPT2 was required for HOXD9-induced PD-L1 upregulation, exANGPT2 or its negative control was co-transfected into NCI-H292 cells with siHOXD9. Results revealed that forced expression of ANGPT2 significantly enhanced total (Fig. 8A) and membrane (Fig. 8B) PD-L1 expression in HOXD9-silenced NCI-H292 cells.

## Discussion

Previously clinic research suggested that HOXD9 level was highly expressed in the cancerous tissues from NSCLC patients [15]. This means that high HOXD9 expression might be associated with the development of NSCLC. Aberrant expression of HOXD9 was proven to play critical roles in carcinogenesis. HOXD9 expression was elevated in several types of cancers, such as glioblastomas, cervical cancer, colorectal carcinoma, gastric cancer, hepatocellular carcinoma and esophageal squamous cell carcinomas [8–13]. Moreover, HOXD9 silencing induced apoptosis, inhibited proliferation, cell cycle progress, migration and invasion of these tumor cells [8–13]. Consistent with the reported oncogenic role of HOXD9 in other cancers, our study suggested that knockdown of HOXD9 induced proliferative suppression, cell cycle arrest, apoptosis, migratory suppression and invasive repression in two human NSCLC cells. The echinoderm microtubule-associated protein-like 4-anaplastic lymphoma kinase (EML4-ALK) gene rearrangement or epidermal growth factor receptor (EGFR) activating mutations has been shown to drive lung tumorigenesis [29, 30]. However, data from clinical trials showed that NSCLC patients carrying the active EGFR or ALK mutation poorly responded to anti-PD-1/PD-L1 immunotherapy [31]. By using two NSCLC cells (NCI-H292 and NCI-H661) with EGFR and ALK wild-type, we found that the PD-L1 expression was reduced after HOXD9 silencing. Our study manifests a pivotal role of HOXD9 in the malignant biological behaviors of NSCLC cells, and indicates a potential value of HOXD9 in immunotherapy. However, the effect of HOXD9 on tumor microenvironment, and whether HOXD9 acts as a specific molecular for targeted therapy of NSCLC need further exploration.

The cell cycle is a complex event involving cells growth and cell division [32]. Dysregulation of G1 cell cycle progression leads to tumor cell proliferation [33]. A previous study indicated that knockdown of HOXD9 increased the number of cells at the G1 phase by downregulating expression of the G1 checkpoint-related genes [14]. Furthermore, HOXD9 inhibition could enhance the expression of p53 [9], a key player in the cell cycle for repairing DNA damage [34]. In our study conducted in two NSCLC cells, we found that silencing of HOXD9 induces the G1 phase arrest, as well as reduced the expression of G1/S-specific cyclin E and G2/M-specific cyclin B1. Our data revealed the association of HOXD9 with cell cycle progression in NSCLC.

In addition to its role in the cell cycle, HOXD9 was demonstrated to suppress apoptosis of tumor cells [8, 9]. Functional analysis in human gliomas suggested that HOXD9 deletion induced apoptosis via downregulation of anti-apoptotic factor B-cell lymphoma-2 (BCL-2) and upregulation of tumor necrosis factor-related apoptosis-inducing ligand (TRAIL) [8]. Moreover, caspase-3/7 activity was shown to be elevated in HOXD9-silenced glioma cells [8]. Hence, HOXD9 inhibition could trigger caspase-3 activation and subsequent programmed cell death through increasing mitochondrial membrane permeability [35]. It was documented that cleavage of PARP1 by caspase-3 resulted in its inactivation, thereby inhibiting DNA repair and promoting apoptosis [36]. In this study, silencing of HOXD9 enhanced the expression of cleaved caspase-3 and cleaved PARP1 in NSCLC cells, identifying the anti-apoptotic function of HOXD9 in NSCLC.

HOXD9 has been shown to promote cancer development via transcriptional activation of oncogenes, such as RUN and FYVE domain containing 3 (RUFY3), Snail family transcriptional repressor 1 (SNAI1), Sodium channel epithelial 1α subunit (SCNN1A) and Hemicentin 1 (HMCN1) [11, 37–39]. By performing dual-luciferase reporter assay, we identified that ANGPT2 was a novel target of HOXD9 in NSCLC cells. It is generally believed that ANGPT2, as an antagonist of ANGPT1, competitively binds to its specific tyrosine kinase receptors-2 (Tie-2) receptors and blocks the vascular-stabilizing effect of Ang-1, leading to sustained neovascularization in tumor tissues [40]. Previous studies have revealed that ANGPT2 expression is elevated in various tumors, such as lung cancer [41], breast cancer [42], and gastric cancer [43]. High expression of ANGPT2 promotes tumor angiogenesis, growth, invasion and metastasis [44]. More importantly, blocking ANGPT2 can effectively improve cancer immunotherapy [45]. Therefore, intervention targeting the ANGPT2 is considered as one of the therapeutic measures for tumors. In the present study, we noted that abnormal expression of ANGPT2 significantly regulated HOXD9-mediated malignant biological behaviors of NSCLC cells and PD-L1 upregulation. Hence, these results indicate that contribution of HOXD9 in lung tumorigenesis may be associated with ANGPT2.

In summary, our findings demonstrate that HOXD9 induces cell proliferation, cell cycle progression, apoptosis suppression, migration and invasion, as well as enhances PD-L1 expression of NSCLC cells via trans-activation of ANGPT2. These indicate that HOXD9 may function as an oncogene in NSCLC. However, a limitation of the current study is the lack of in vivo data. Additional experiments with animal models will be needed in future studies. In addition, it is necessary to evaluate clinicopathological correlation between HOXD9 expression and NSCLC patients in further studies to support our conclusion.

## Supplementary Information

**Additional file 1. **

## Data Availability

The data that support the findings of this study are available from the corresponding author upon reasonable request.
