# Supplementary figures and images for: Homeobox D9 drives the malignant phenotypes and enhances the Programmed death ligand-1 expression in non-small cell lung cancer cells via binding to Angiopoietin-2 promoter
Source: World J Surg Oncol. 2023 Mar 13;21:93. doi: 10.1186/s12957-023-02969-z (PMC10009994; doi:10.1186/s12957-023-02969-z)

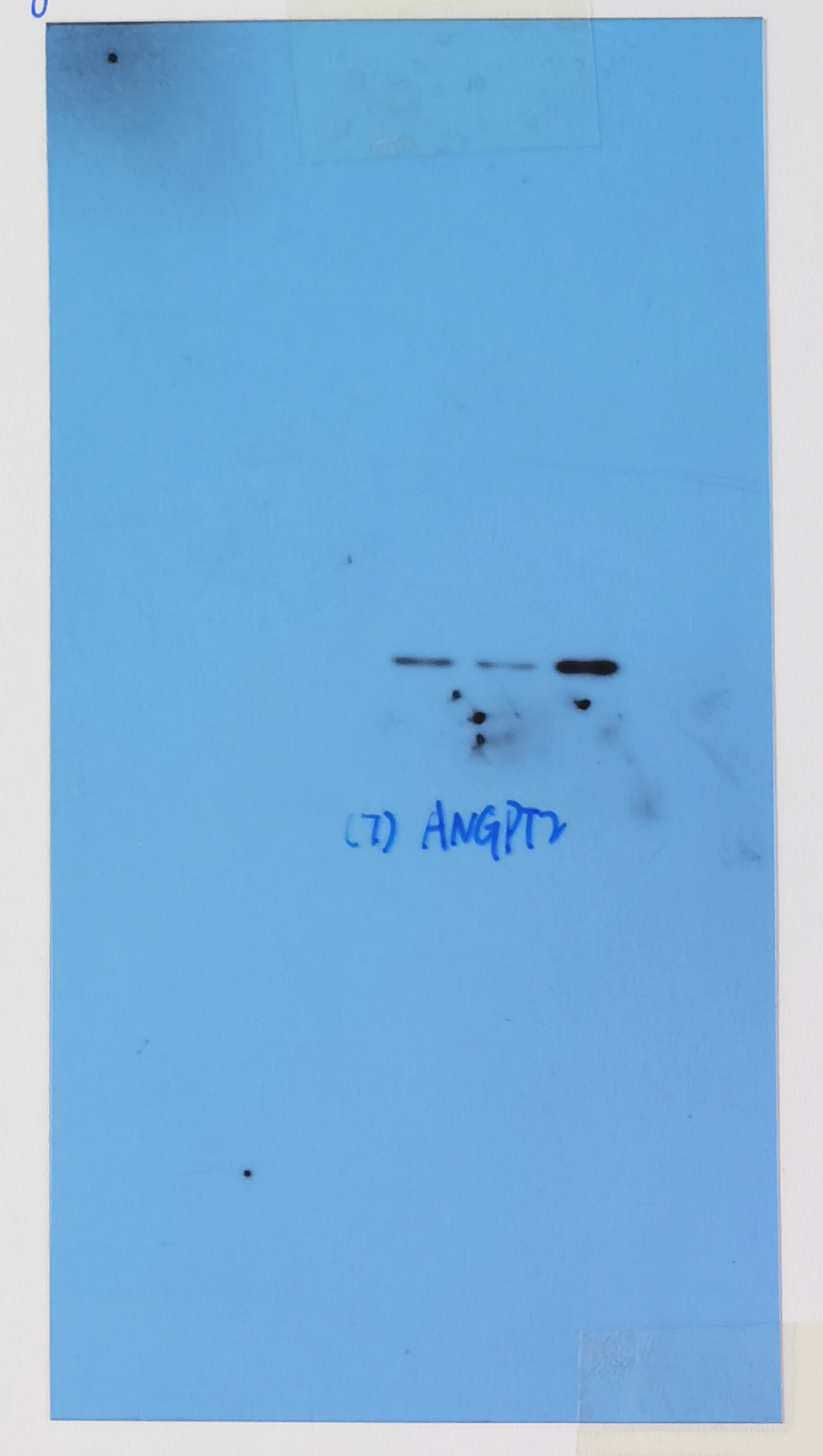

Supplement: Supplementary file 1 — Additional file 1. [file 12957_2023_2969_MOESM1_ESM.zip › ANGPT2 (fig3C left).tif]

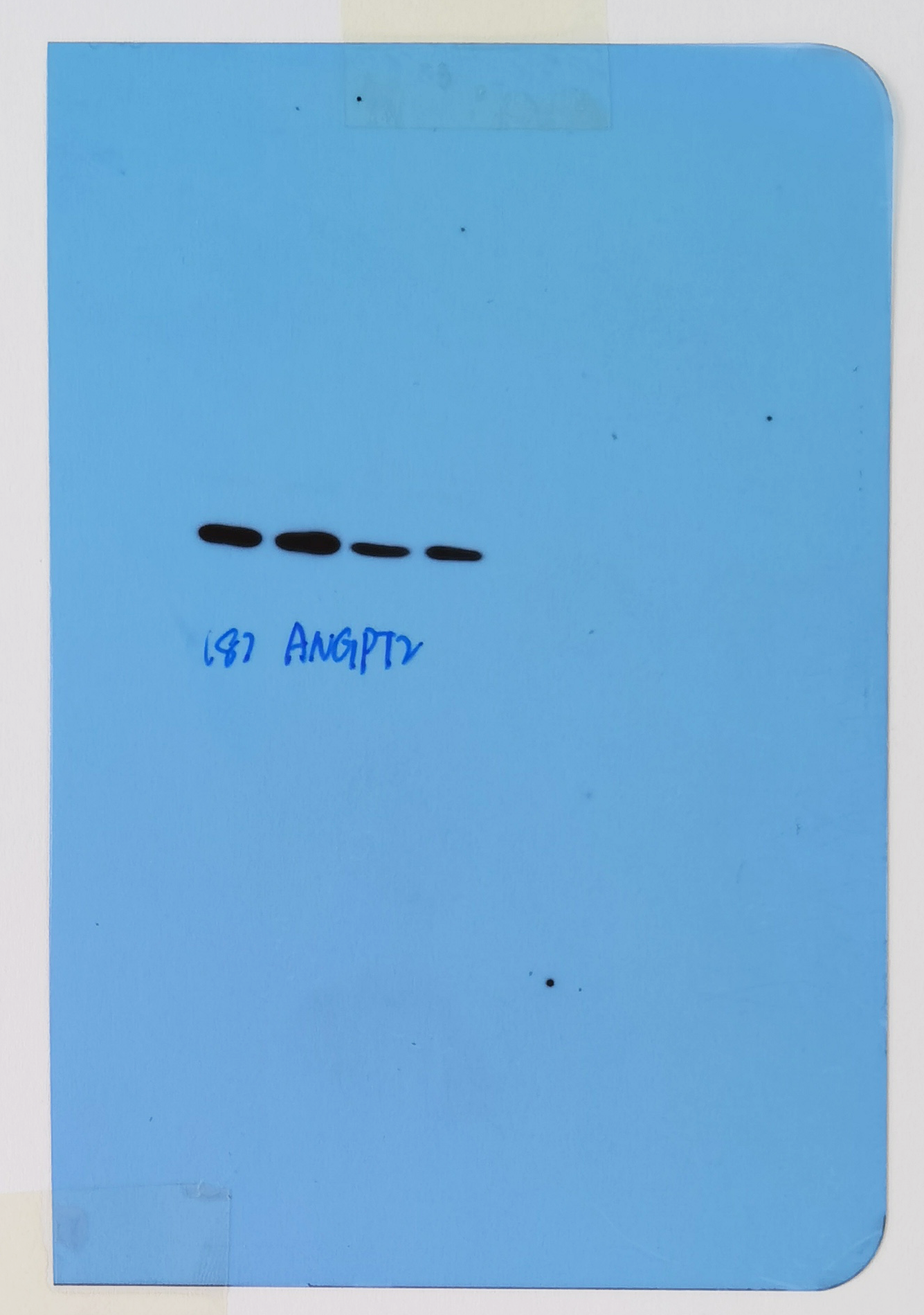

Supplement: Supplementary file 1 — Additional file 1. [file 12957_2023_2969_MOESM1_ESM.zip › ANGPT2 (fig3C right).tif]

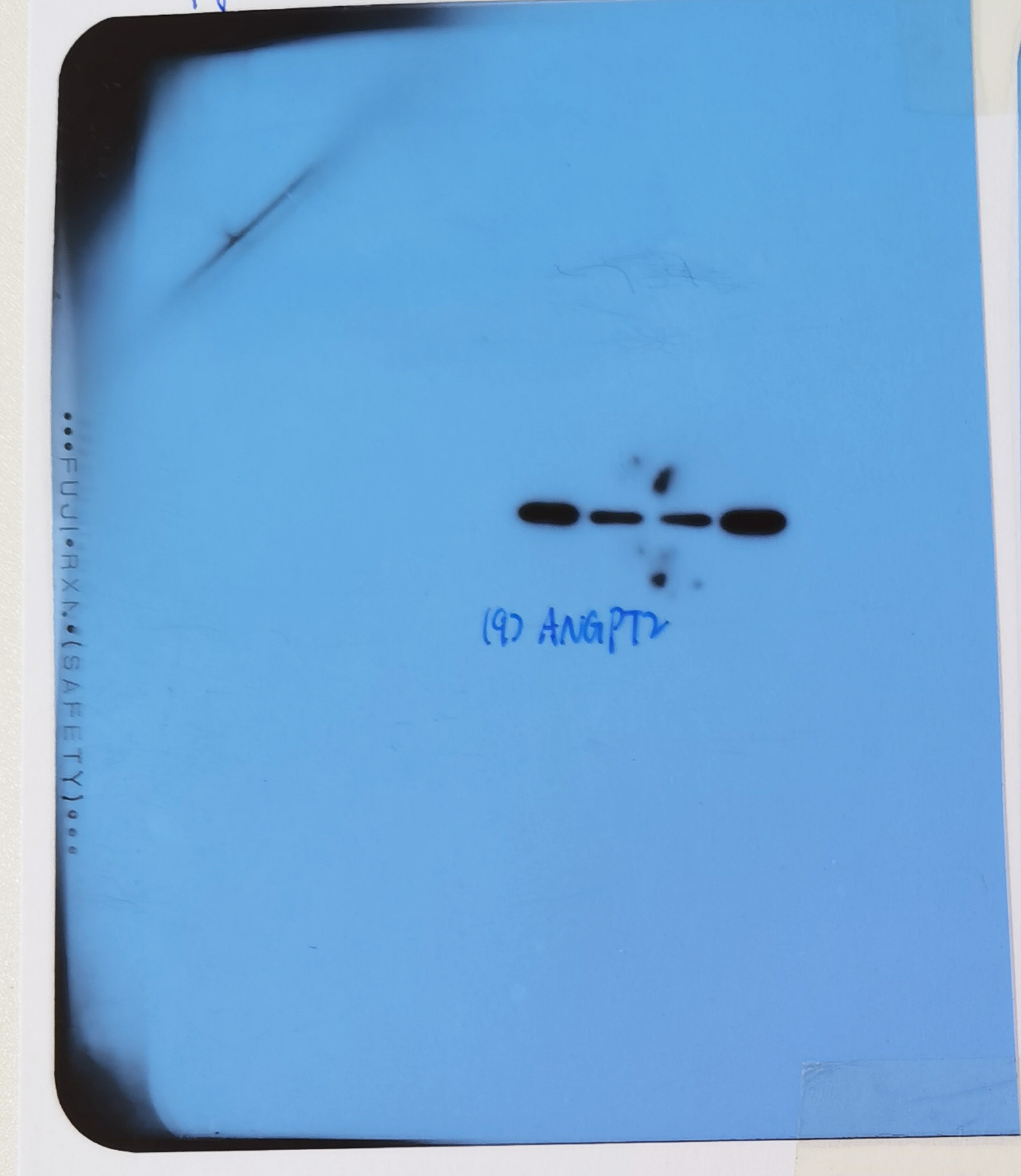

Supplement: Supplementary file 1 — Additional file 1. [file 12957_2023_2969_MOESM1_ESM.zip › ANGPT2 (fig4B left).tif]

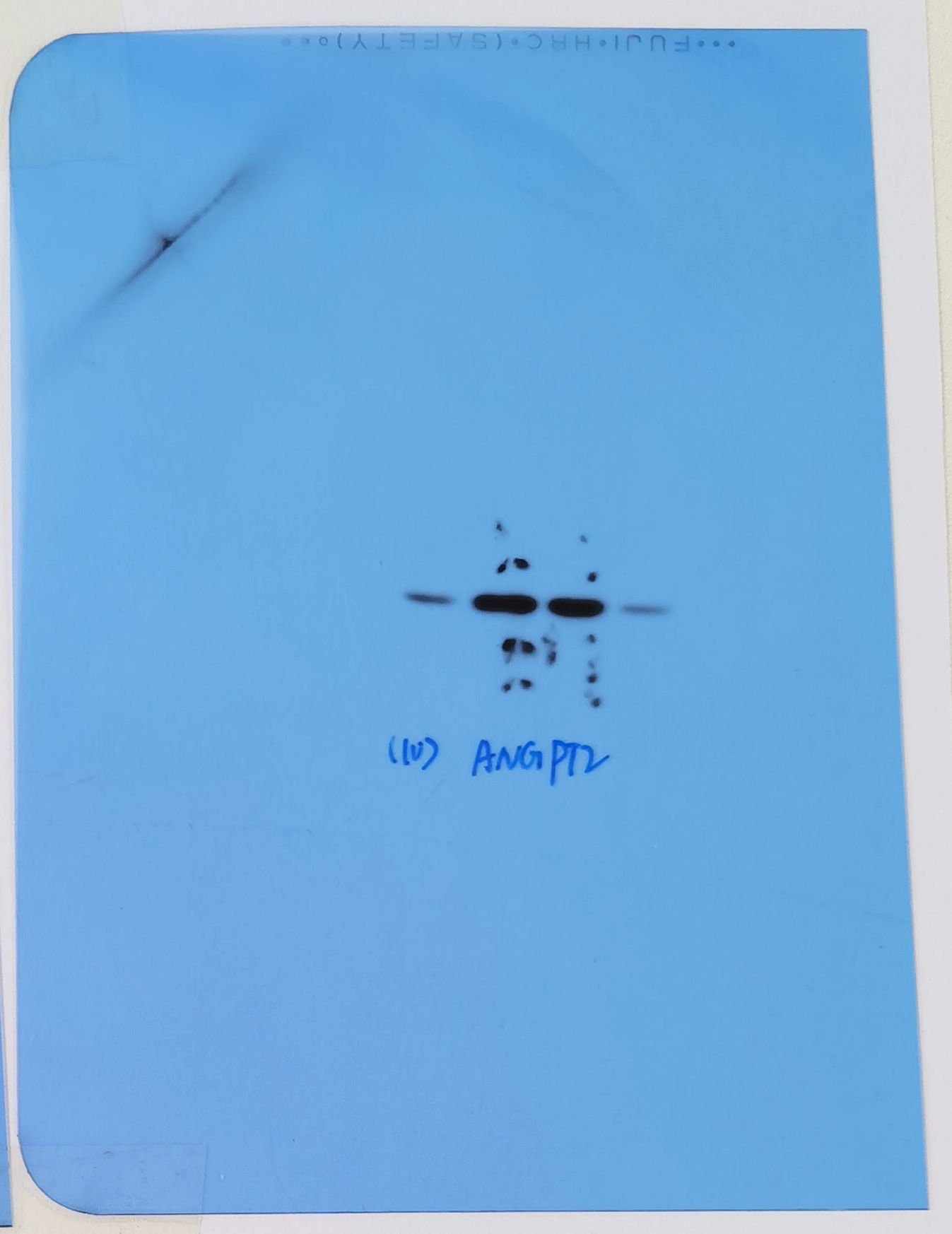

Supplement: Supplementary file 1 — Additional file 1. [file 12957_2023_2969_MOESM1_ESM.zip › ANGPT2 (fig4B right).tif]

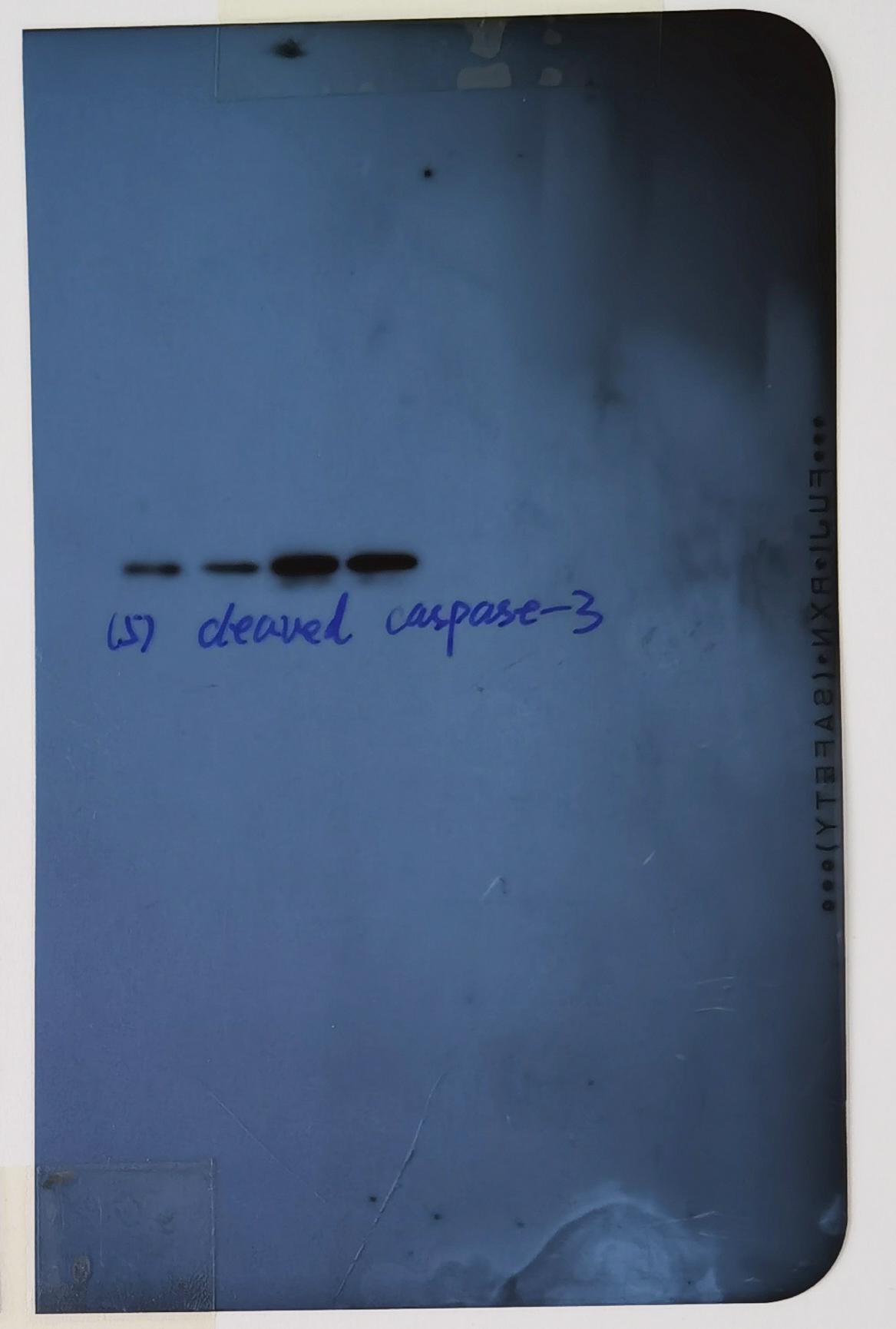

Supplement: Supplementary file 1 — Additional file 1. [file 12957_2023_2969_MOESM1_ESM.zip › cleaved caspase-3 (fig2G upper).tif]

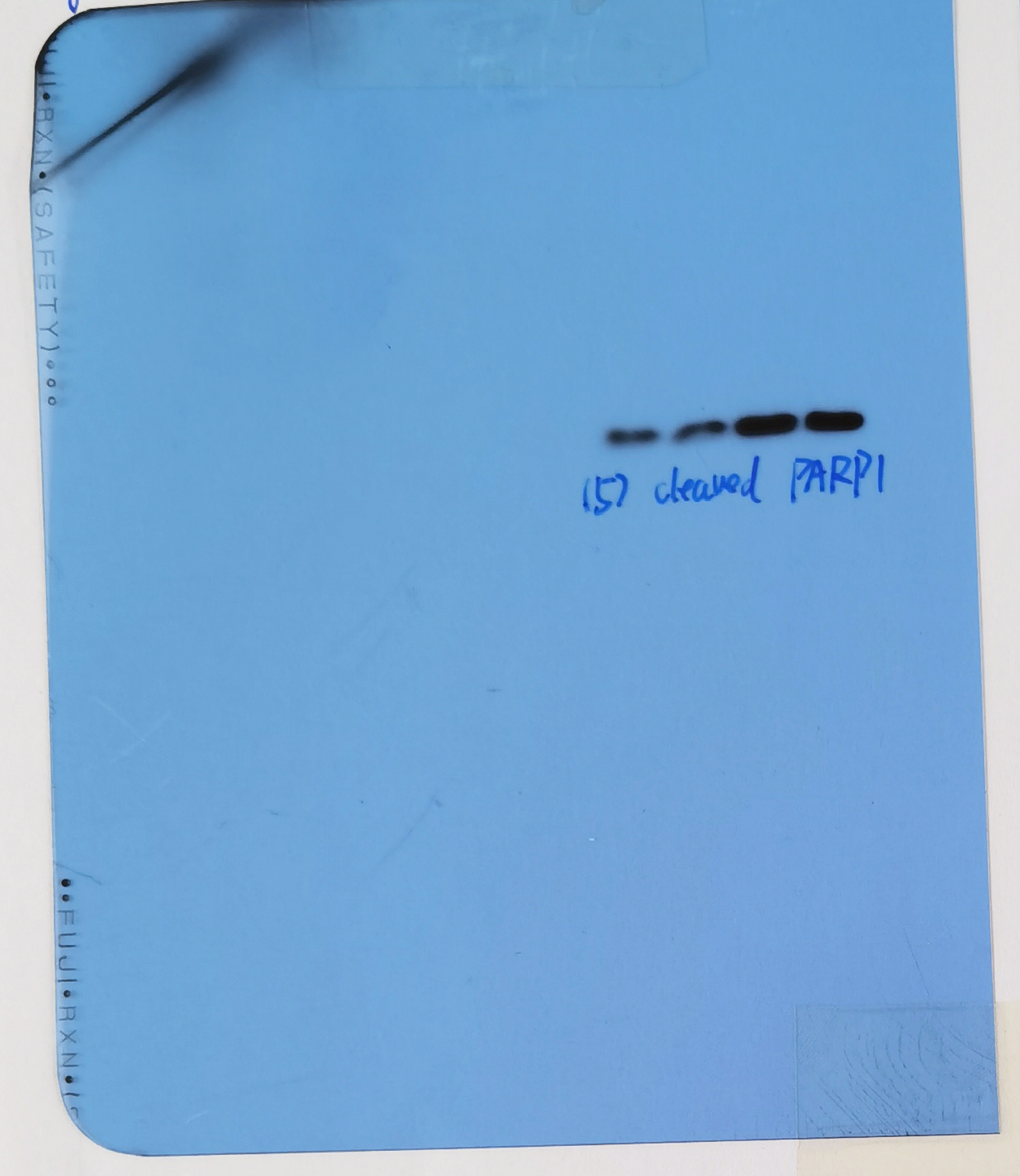

Supplement: Supplementary file 1 — Additional file 1. [file 12957_2023_2969_MOESM1_ESM.zip › cleaved PARP1 (fig2G upper).tif]

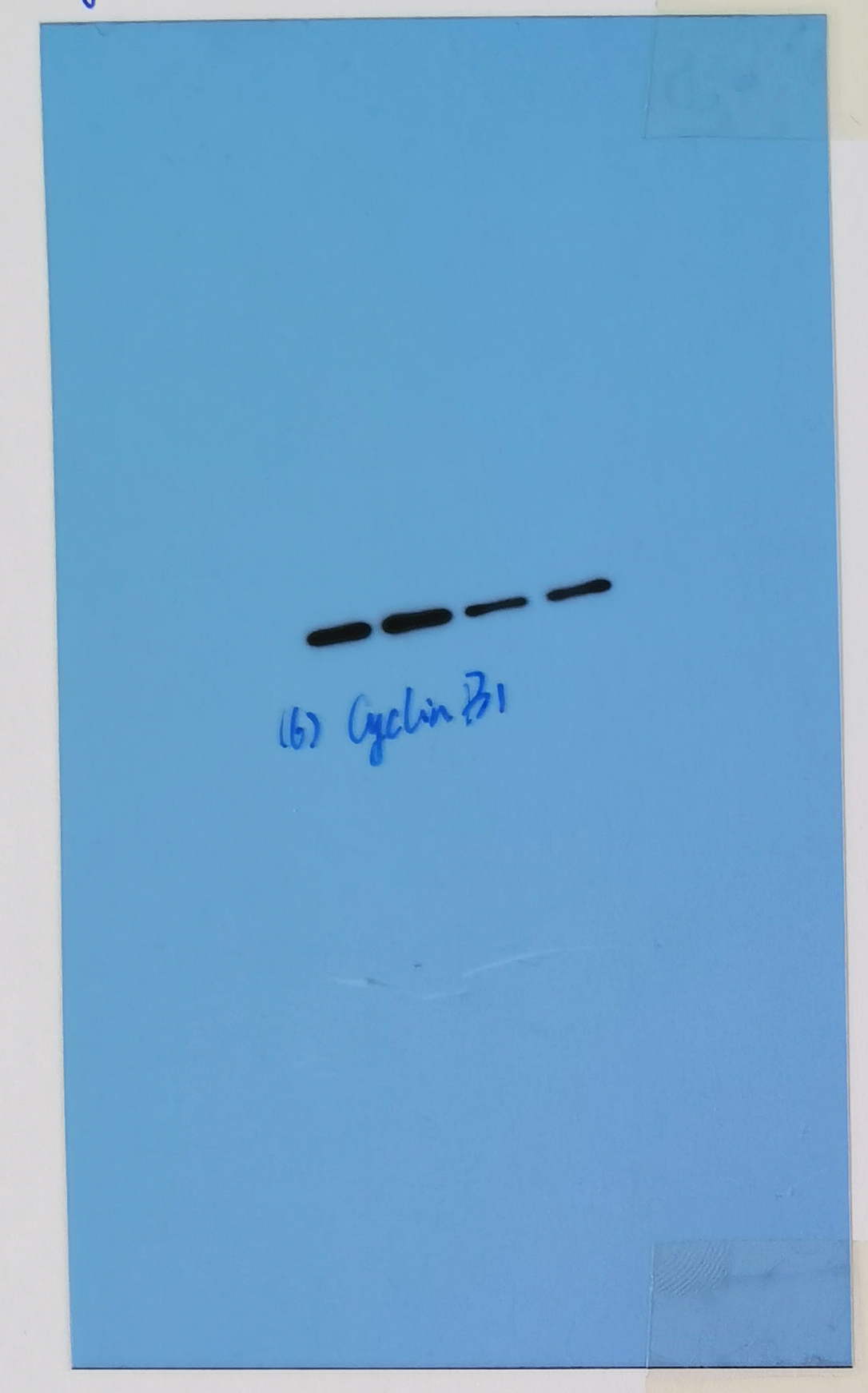

Supplement: Supplementary file 1 — Additional file 1. [file 12957_2023_2969_MOESM1_ESM.zip › Cyclin B1 (fig2G bottom).tif]

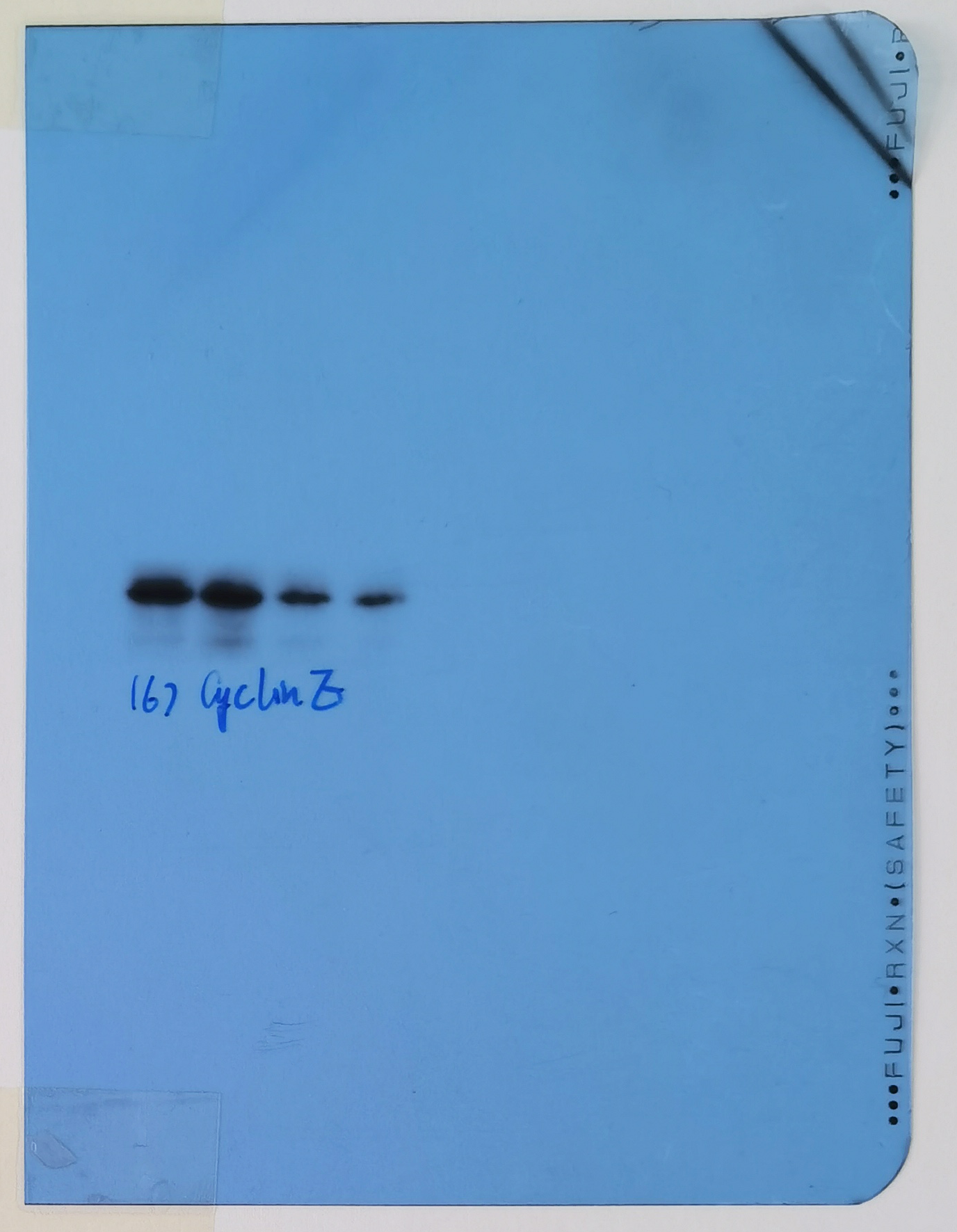

Supplement: Supplementary file 1 — Additional file 1. [file 12957_2023_2969_MOESM1_ESM.zip › Cyclin E (fig2G bottom).tif]

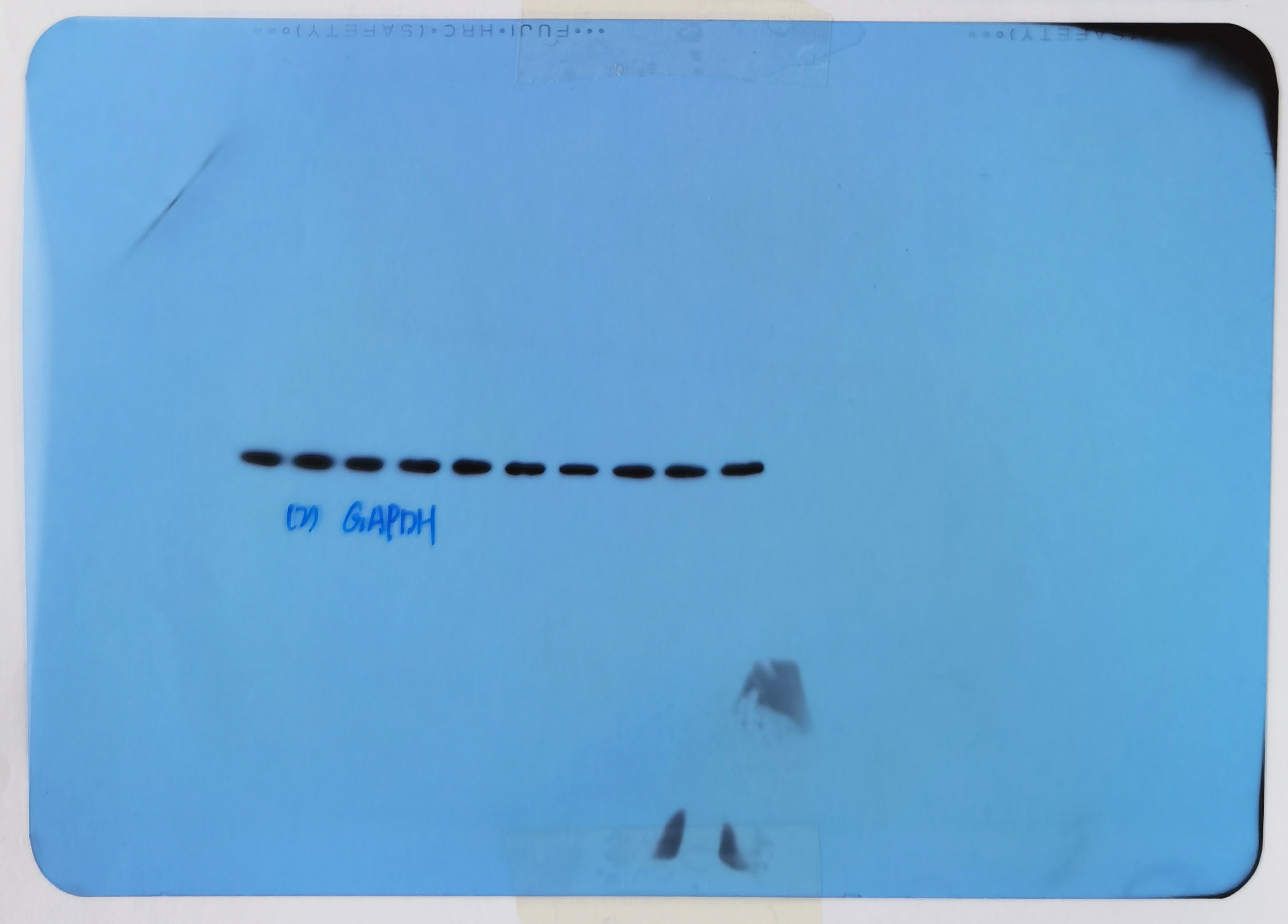

Supplement: Supplementary file 1 — Additional file 1. [file 12957_2023_2969_MOESM1_ESM.zip › GAPDH (fig1B bottom).tif]

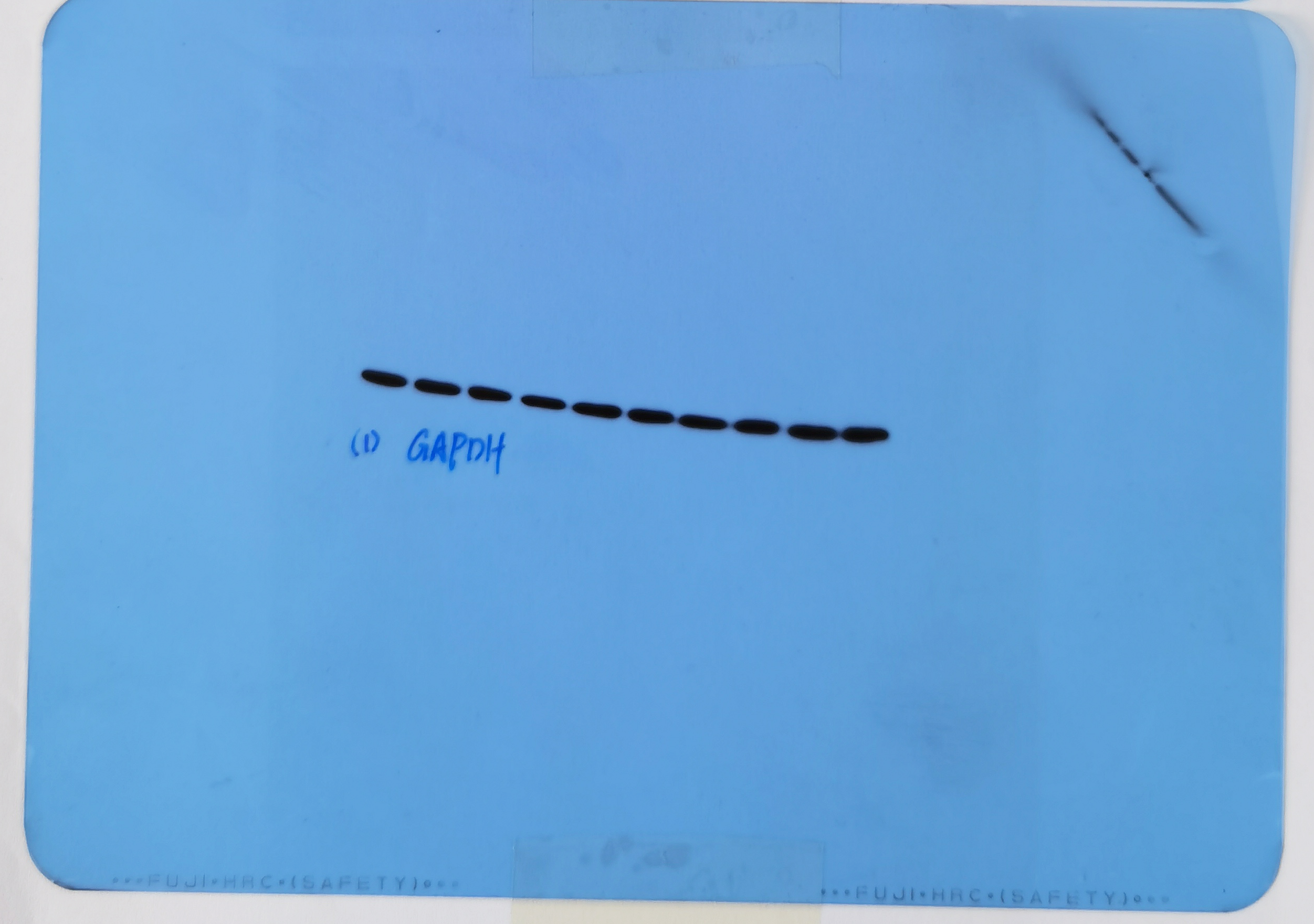

Supplement: Supplementary file 1 — Additional file 1. [file 12957_2023_2969_MOESM1_ESM.zip › GAPDH (fig1B upper).tif]

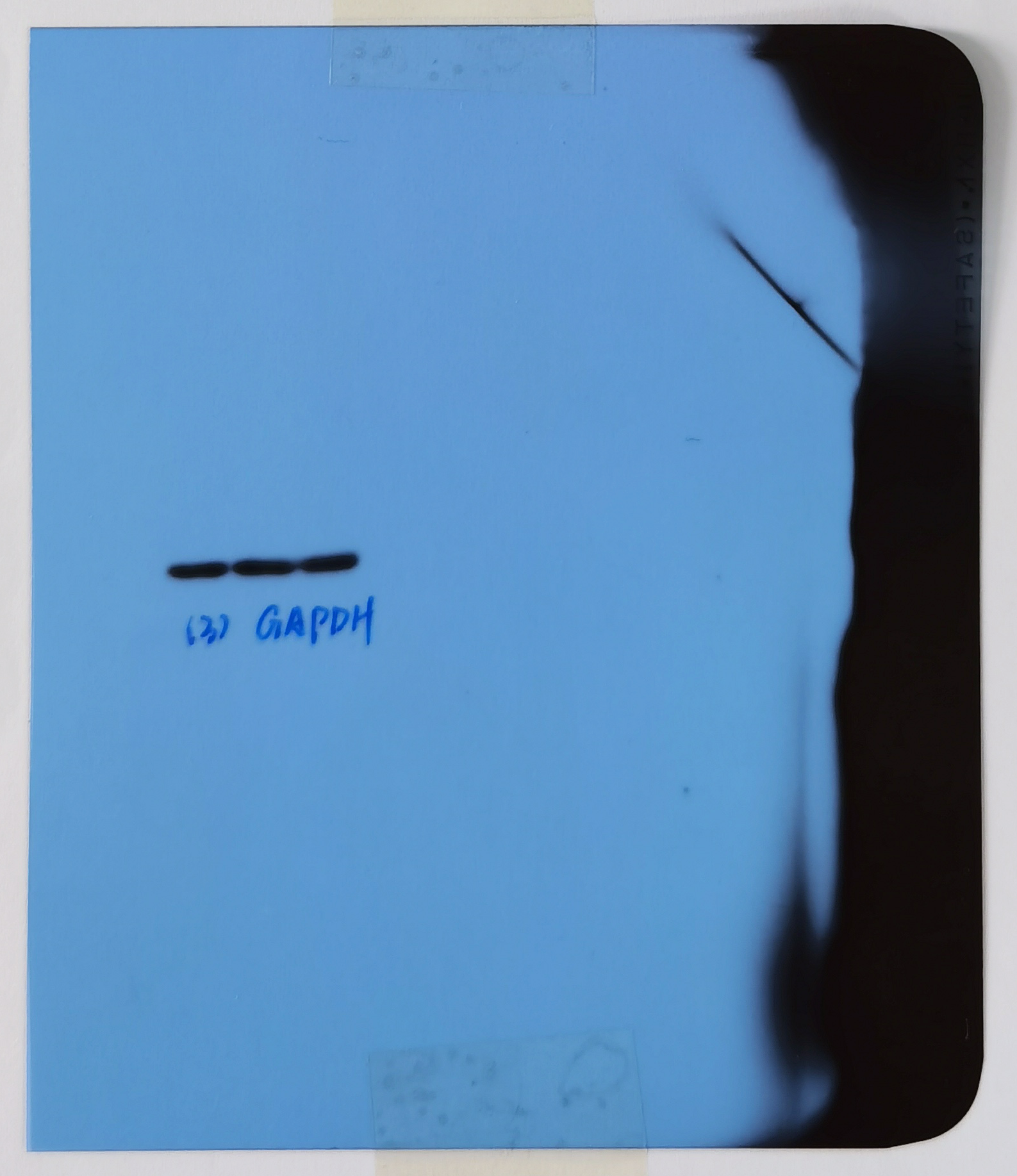

Supplement: Supplementary file 1 — Additional file 1. [file 12957_2023_2969_MOESM1_ESM.zip › GAPDH (fig2B left).tif]

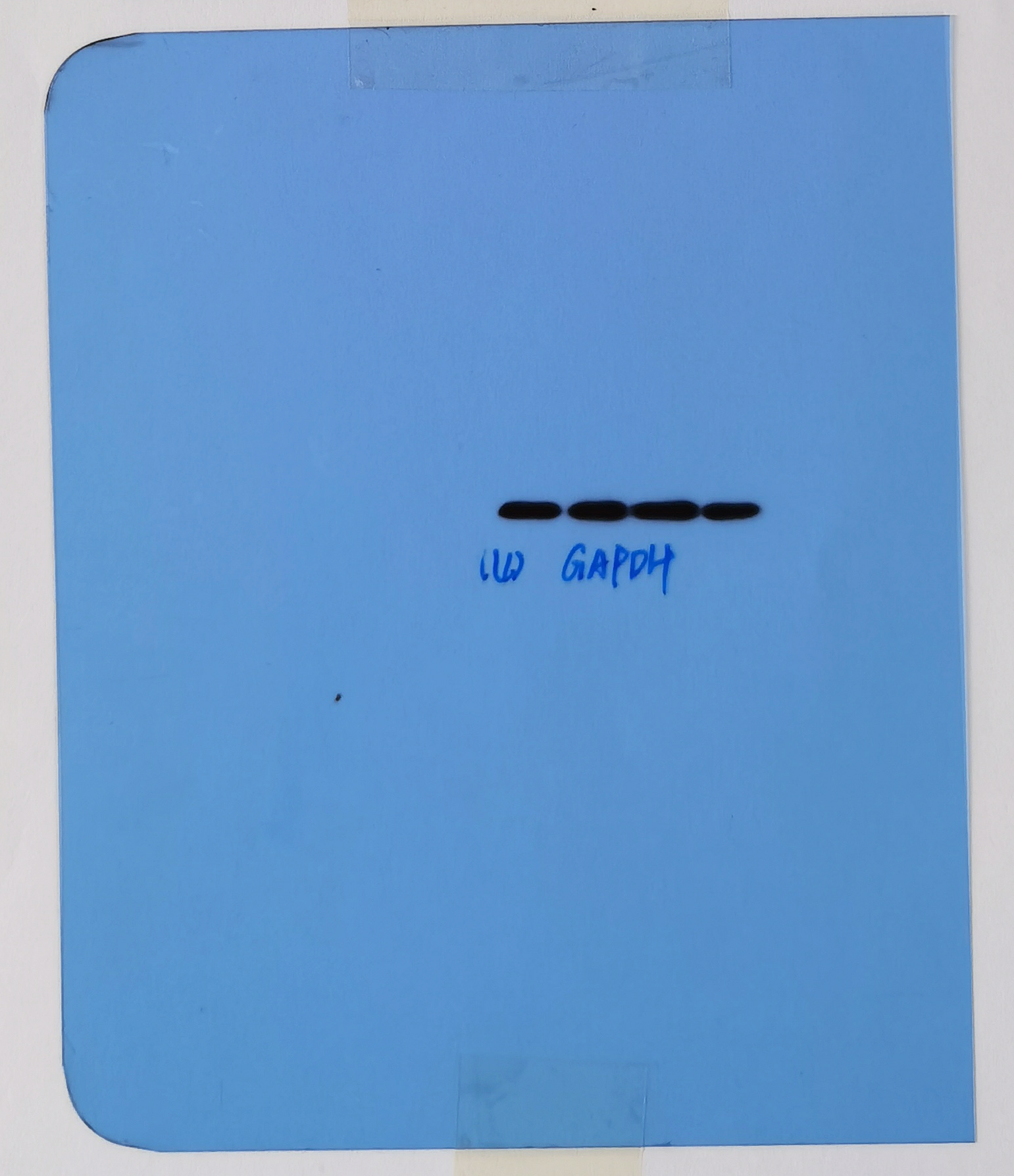

Supplement: Supplementary file 1 — Additional file 1. [file 12957_2023_2969_MOESM1_ESM.zip › GAPDH (fig2B right).tif]

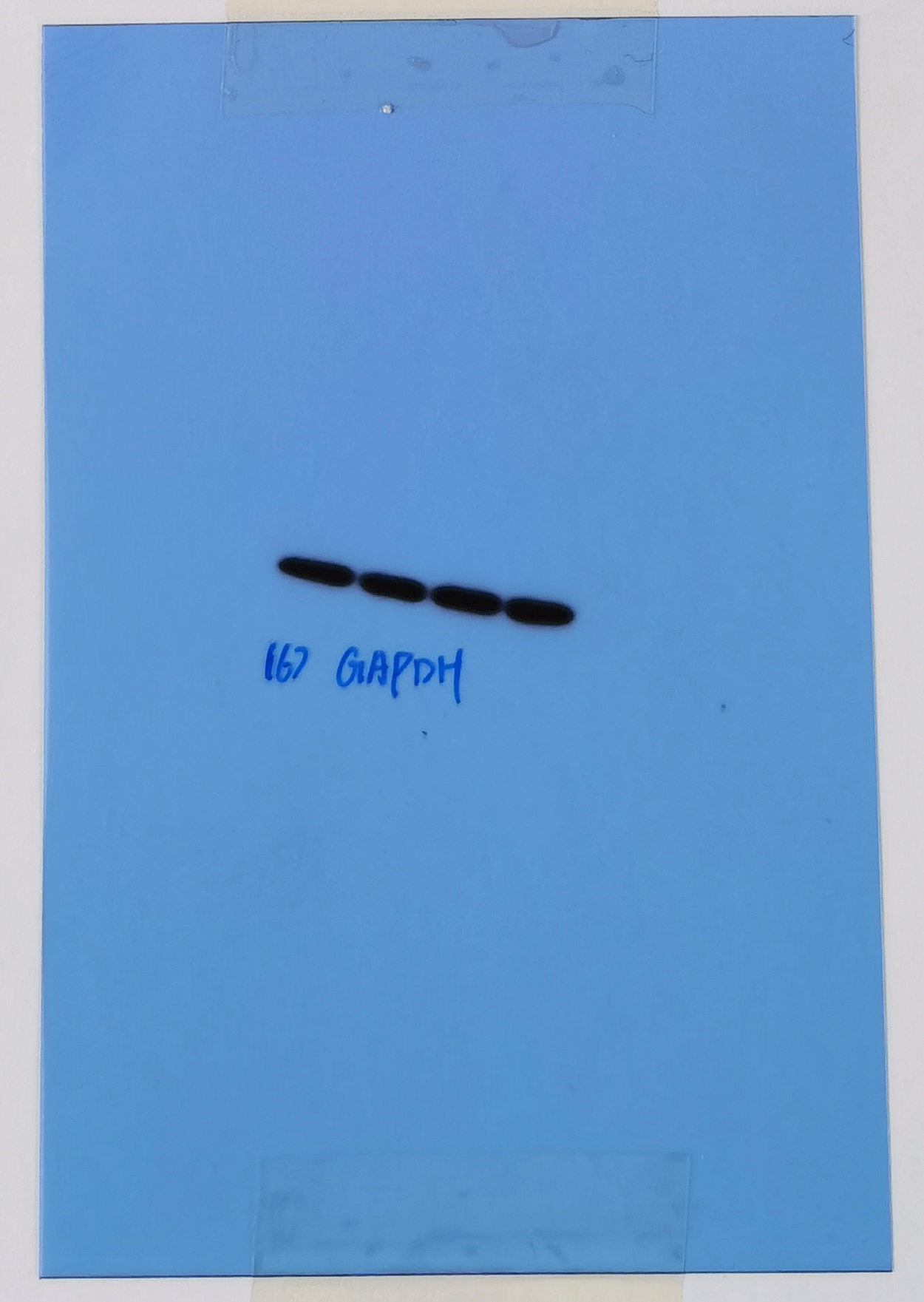

Supplement: Supplementary file 1 — Additional file 1. [file 12957_2023_2969_MOESM1_ESM.zip › GAPDH (fig2G bottom).tif]

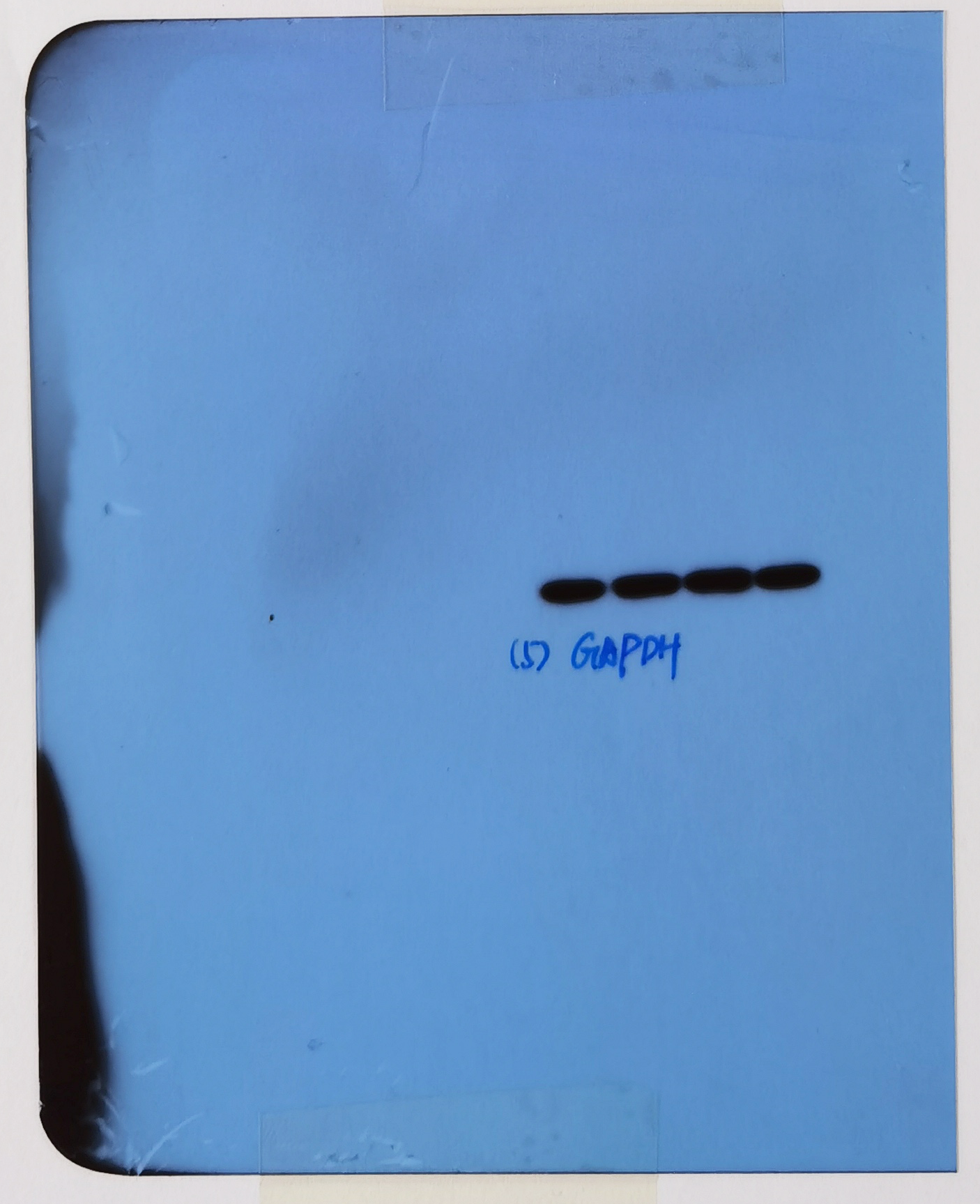

Supplement: Supplementary file 1 — Additional file 1. [file 12957_2023_2969_MOESM1_ESM.zip › GAPDH (fig2G upper).tif]

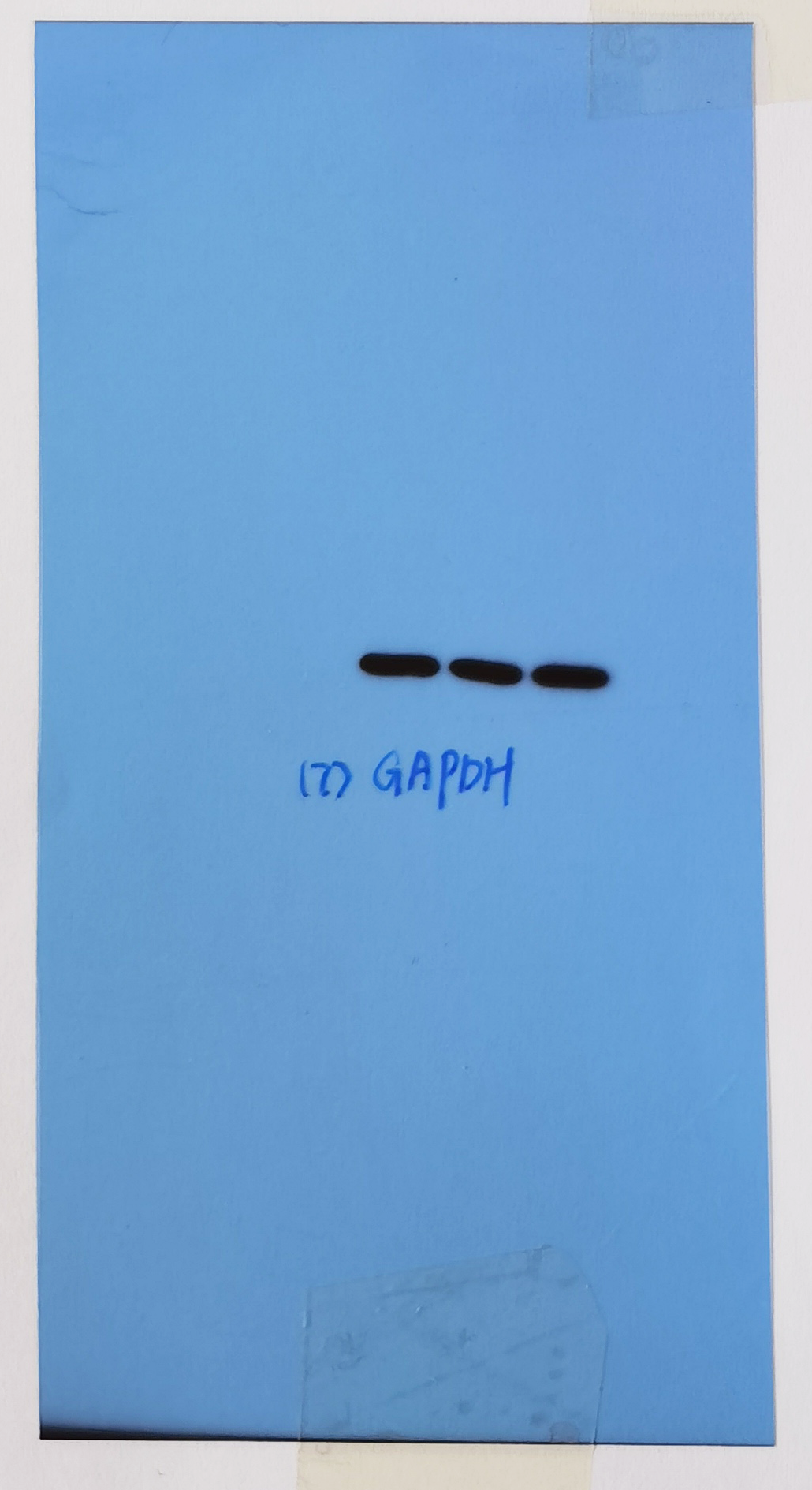

Supplement: Supplementary file 1 — Additional file 1. [file 12957_2023_2969_MOESM1_ESM.zip › GAPDH (fig3C left).tif]

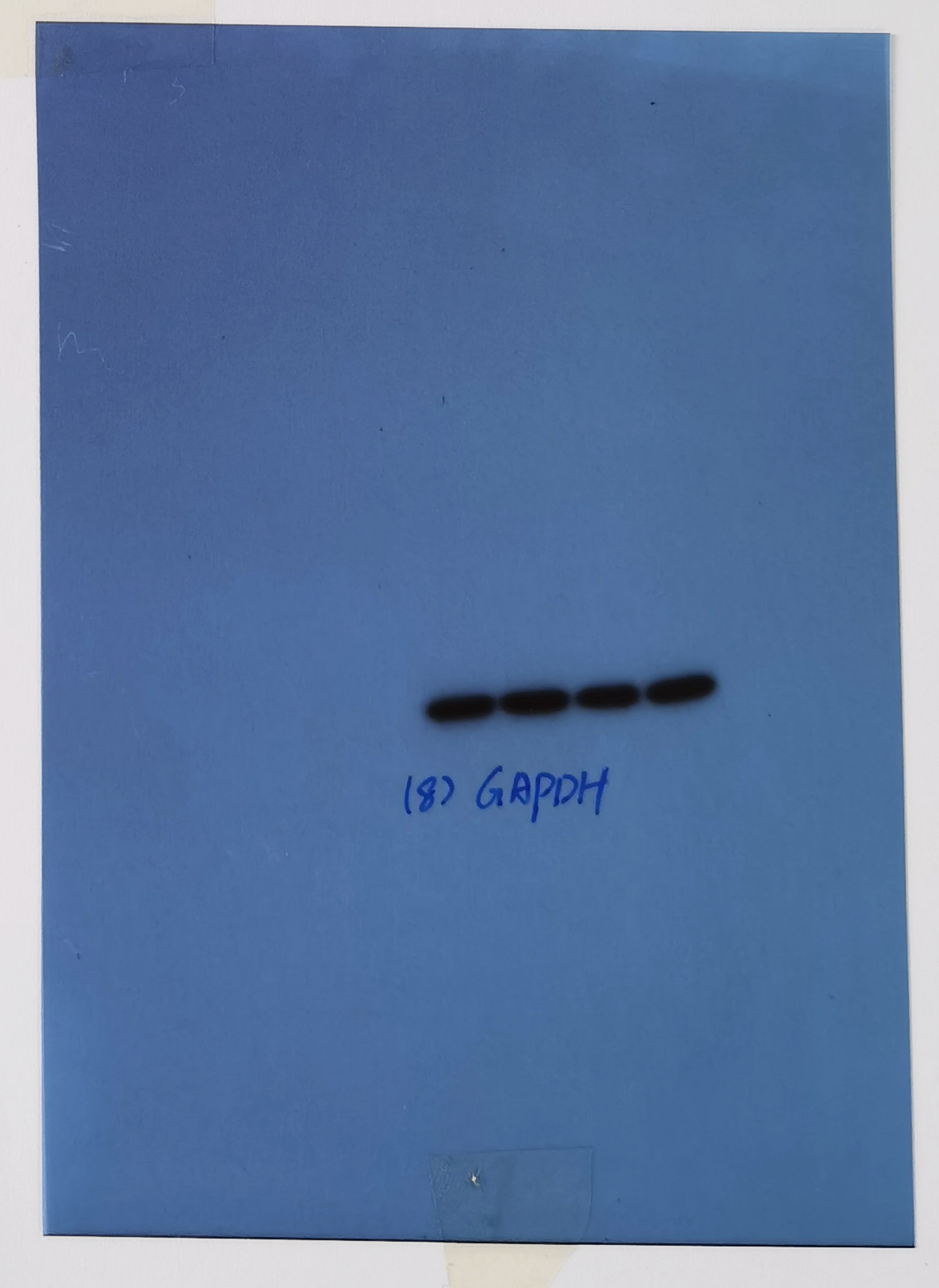

Supplement: Supplementary file 1 — Additional file 1. [file 12957_2023_2969_MOESM1_ESM.zip › GAPDH (fig3C right).tif]

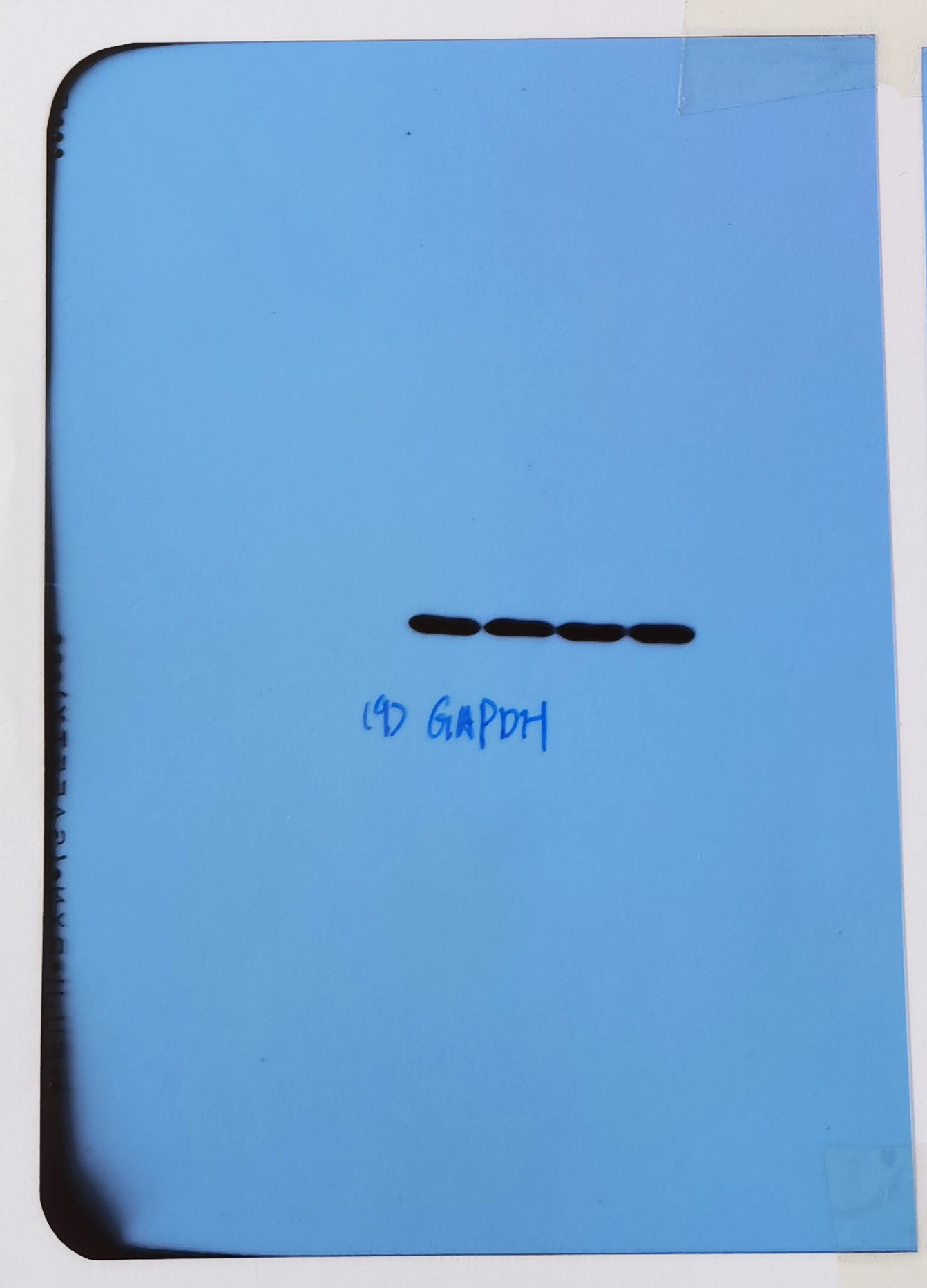

Supplement: Supplementary file 1 — Additional file 1. [file 12957_2023_2969_MOESM1_ESM.zip › GAPDH (fig4B left).tif]

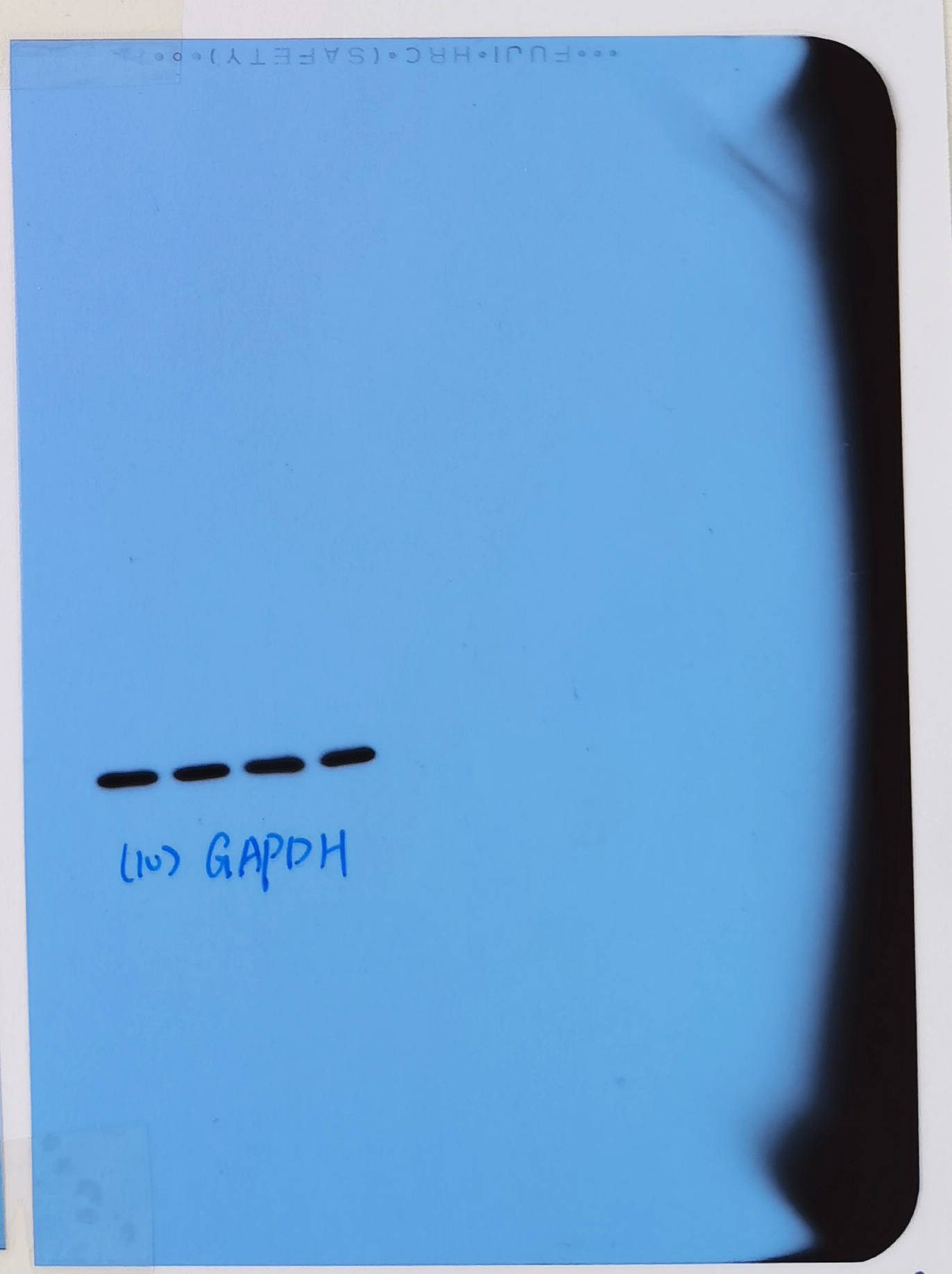

Supplement: Supplementary file 1 — Additional file 1. [file 12957_2023_2969_MOESM1_ESM.zip › GAPDH (fig4B right).tif]

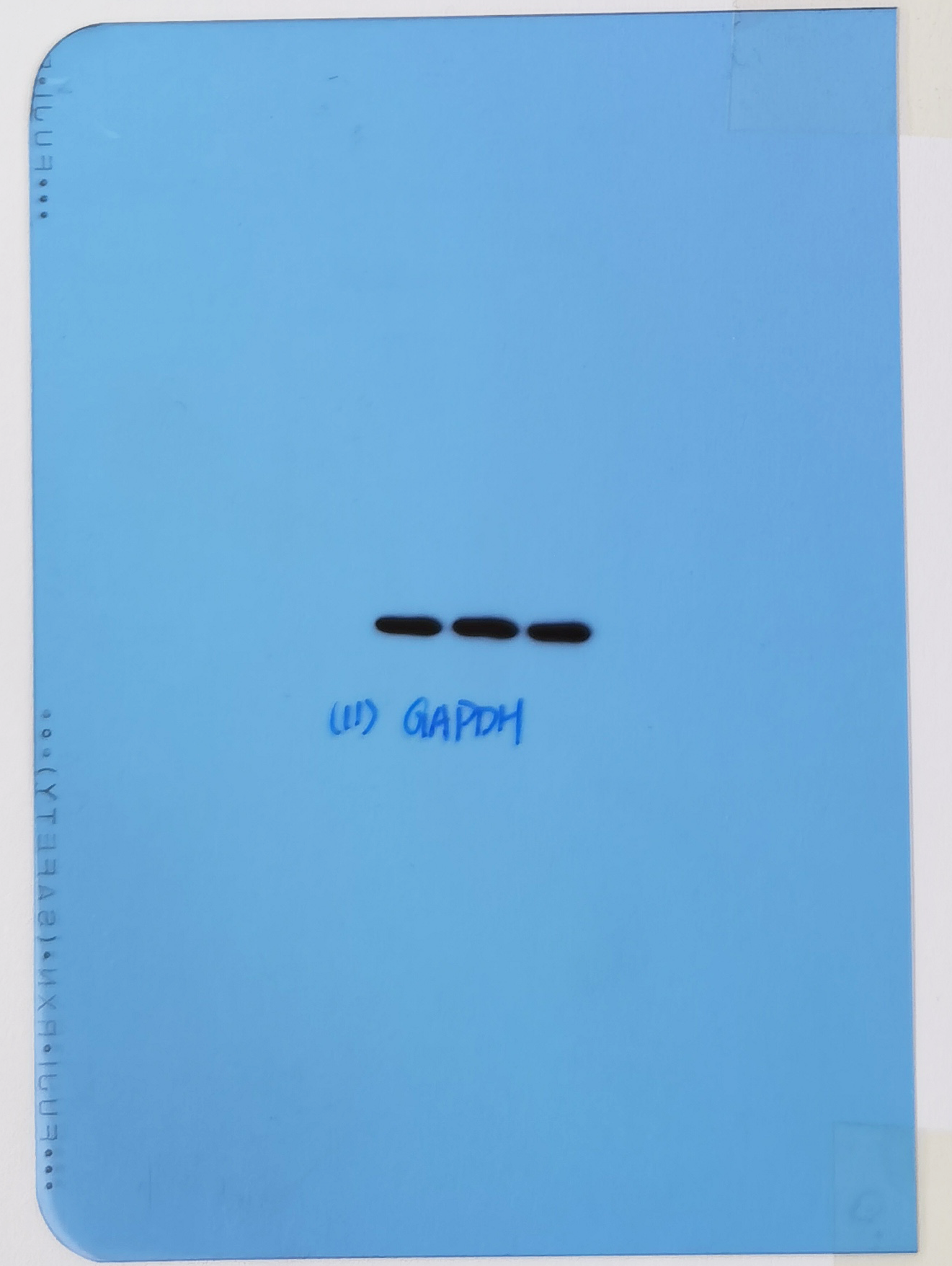

Supplement: Supplementary file 1 — Additional file 1. [file 12957_2023_2969_MOESM1_ESM.zip › GAPDH (fig5A left).tif]

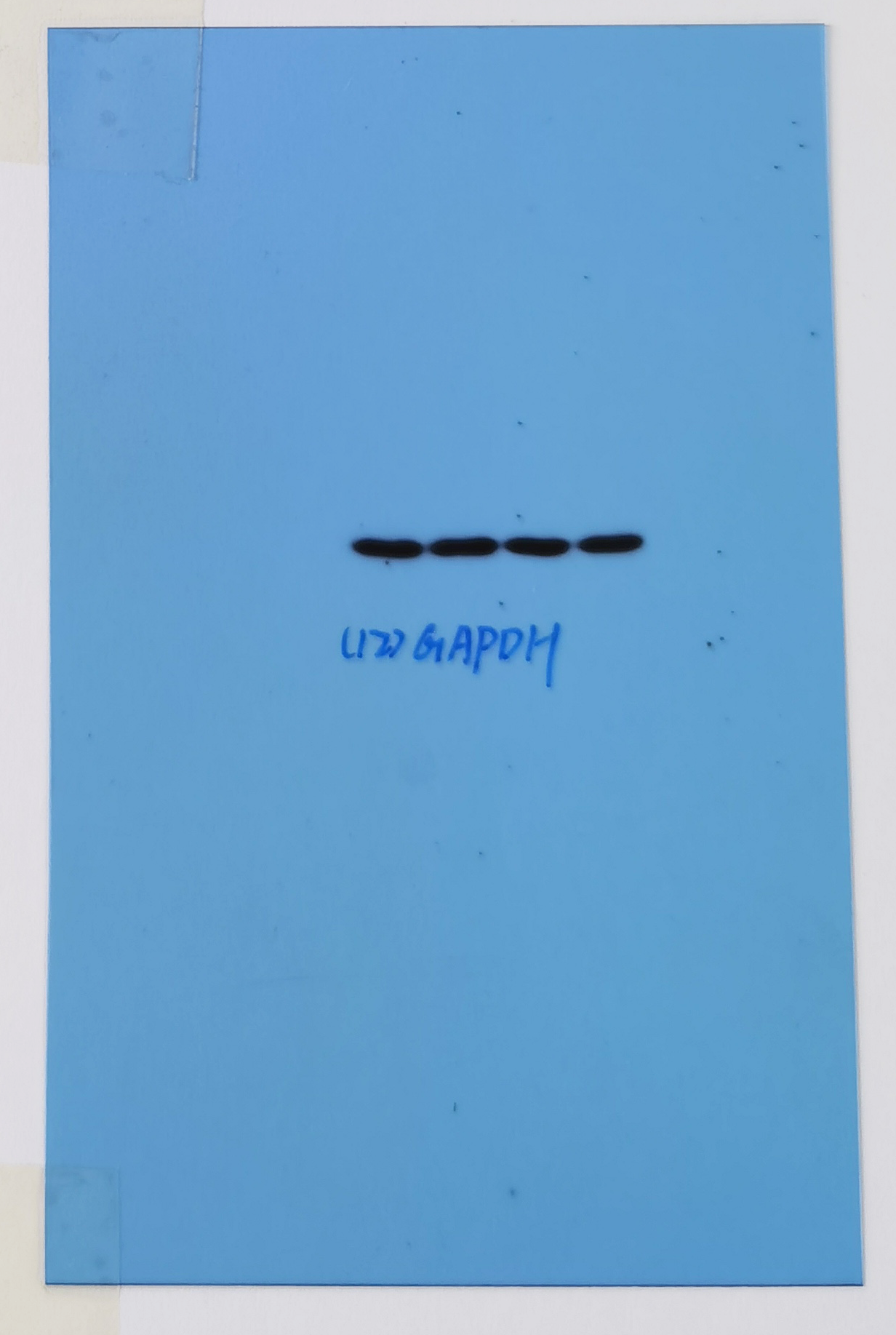

Supplement: Supplementary file 1 — Additional file 1. [file 12957_2023_2969_MOESM1_ESM.zip › GAPDH (fig5A right).tif]

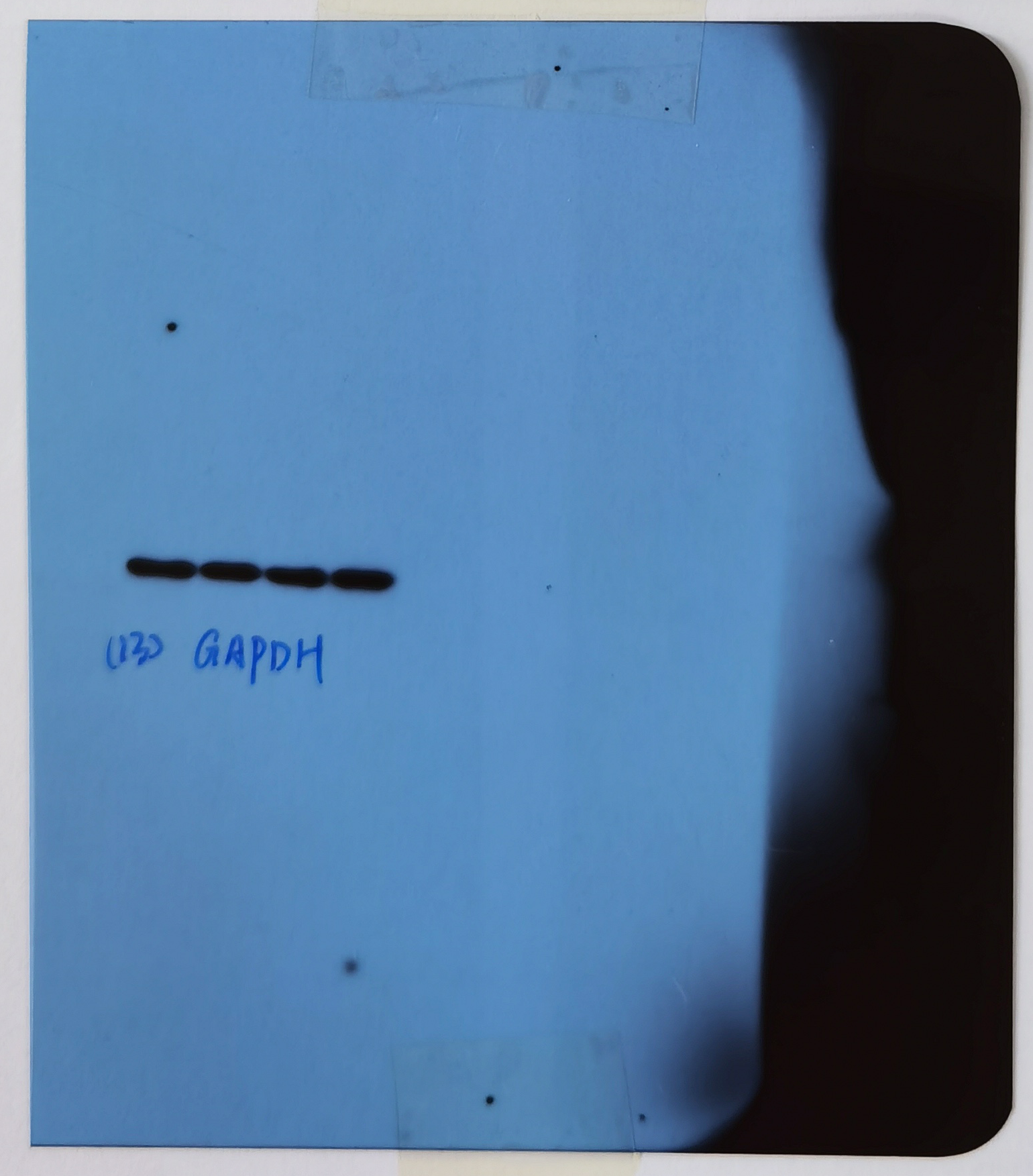

Supplement: Supplementary file 1 — Additional file 1. [file 12957_2023_2969_MOESM1_ESM.zip › GAPDH (fig6A).tif]

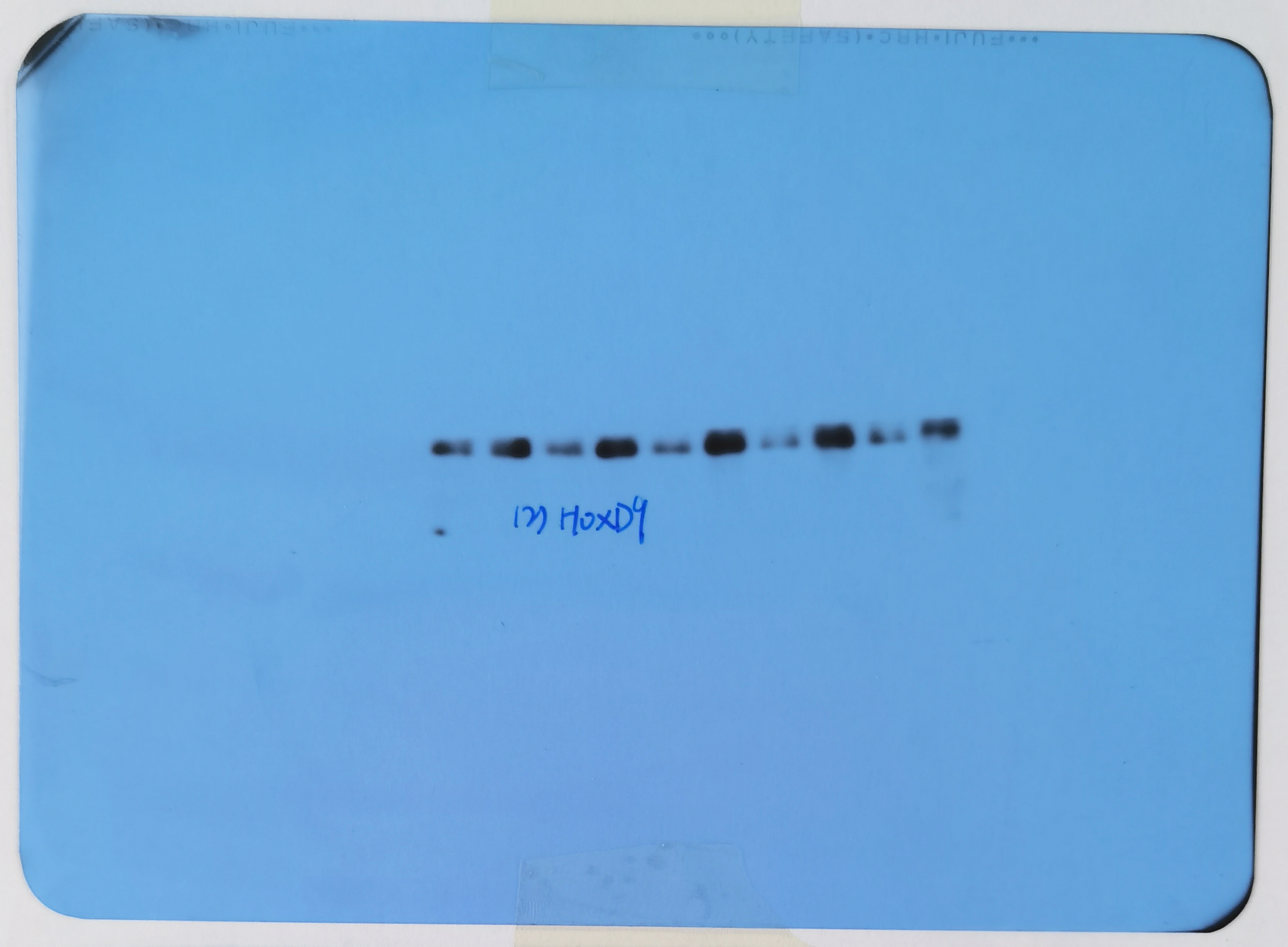

Supplement: Supplementary file 1 — Additional file 1. [file 12957_2023_2969_MOESM1_ESM.zip › HOXD9 (fig1B bottom).tif]

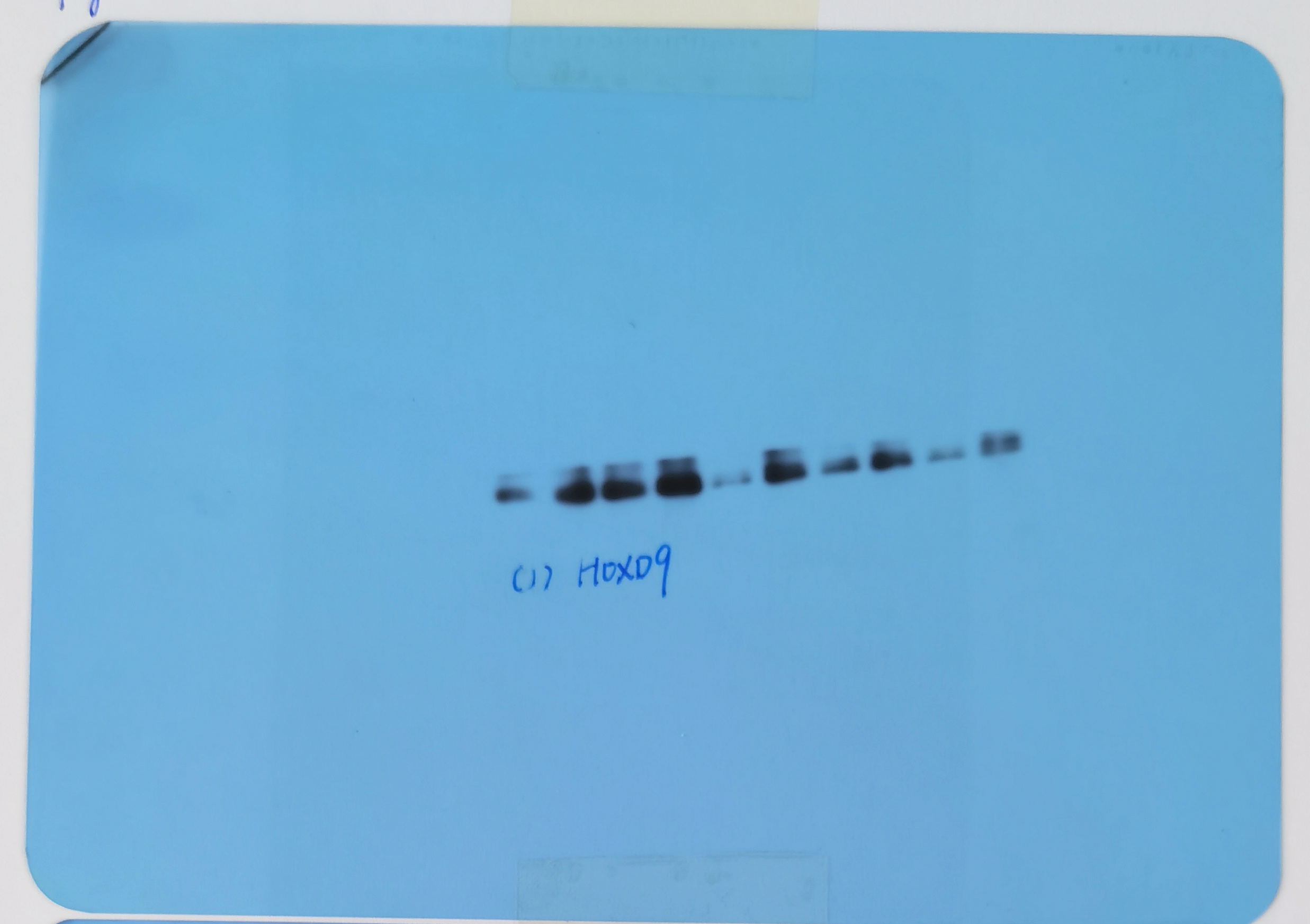

Supplement: Supplementary file 1 — Additional file 1. [file 12957_2023_2969_MOESM1_ESM.zip › HOXD9 (fig1B upper).tif]

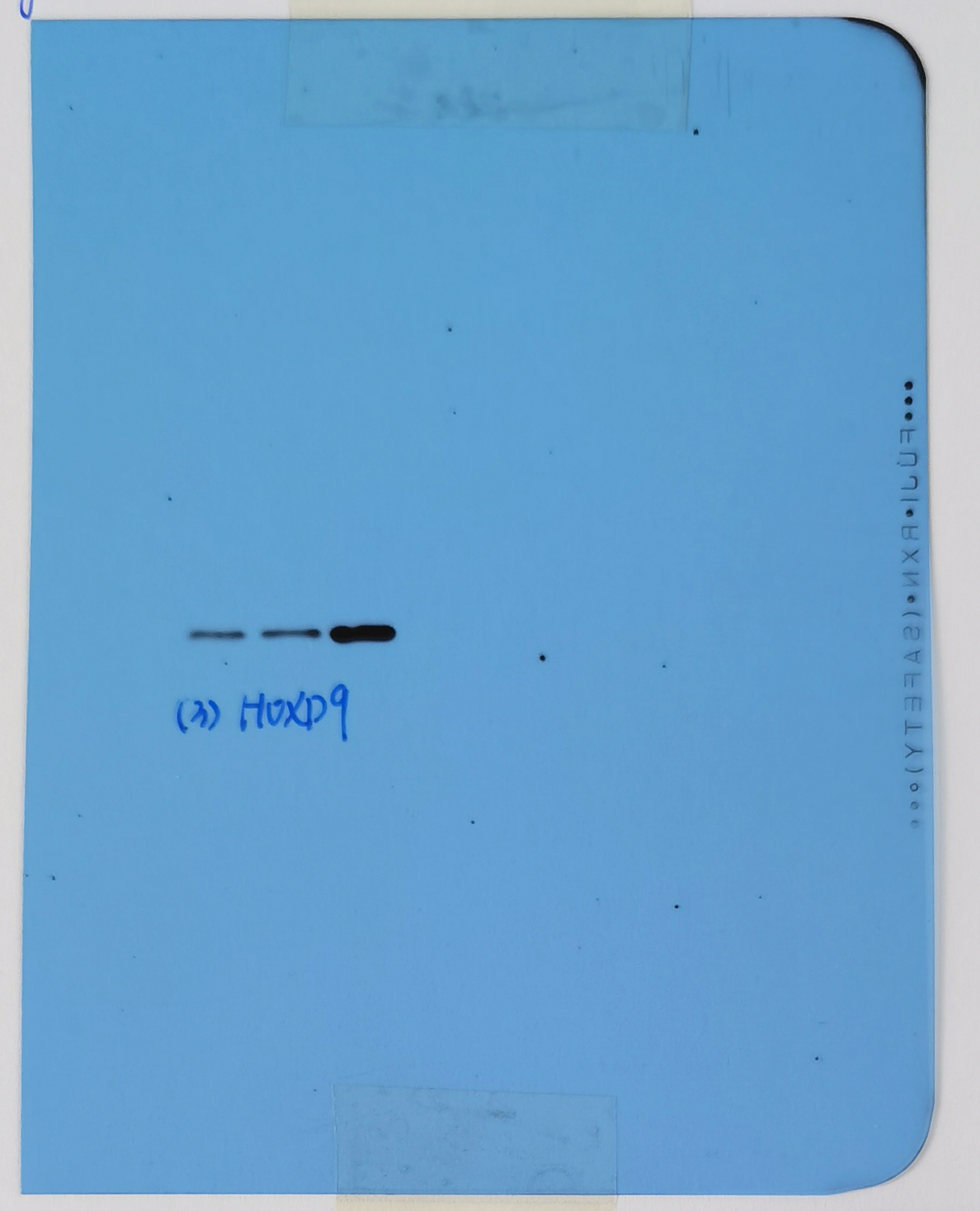

Supplement: Supplementary file 1 — Additional file 1. [file 12957_2023_2969_MOESM1_ESM.zip › HOXD9 (fig2B left).tif]

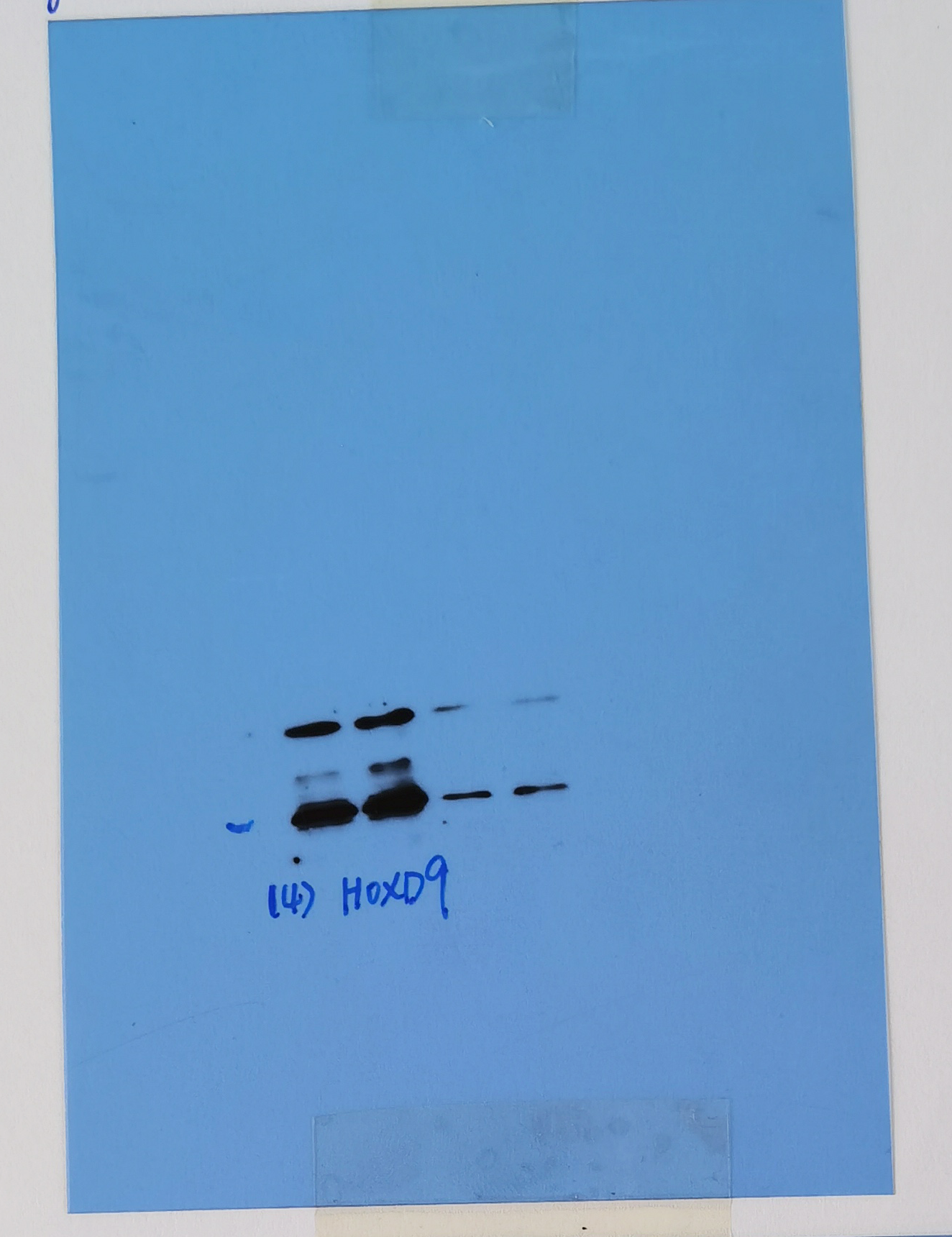

Supplement: Supplementary file 1 — Additional file 1. [file 12957_2023_2969_MOESM1_ESM.zip › HOXD9 (fig2B right).tif]

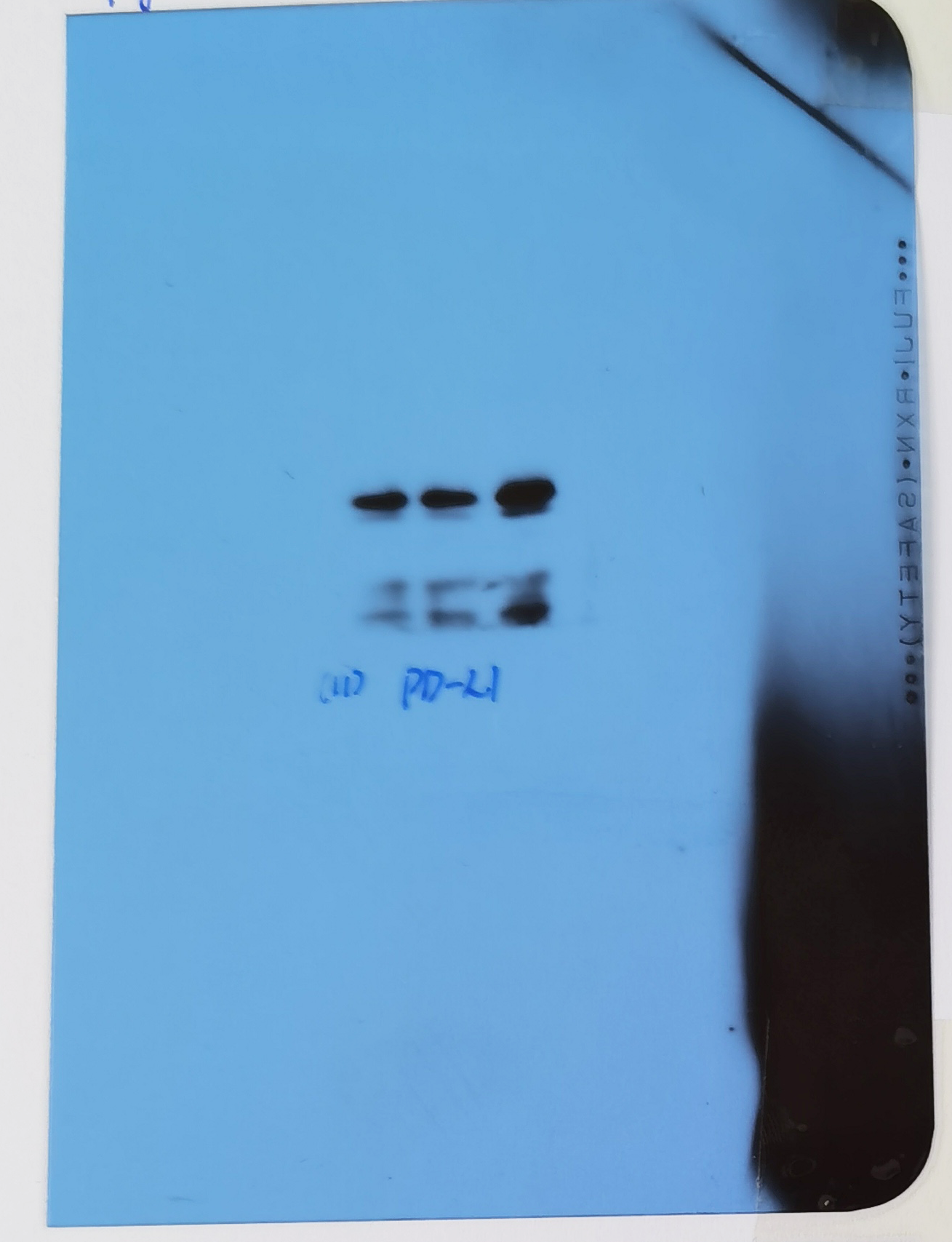

Supplement: Supplementary file 1 — Additional file 1. [file 12957_2023_2969_MOESM1_ESM.zip › PD-L1 (fig5A left).tif]

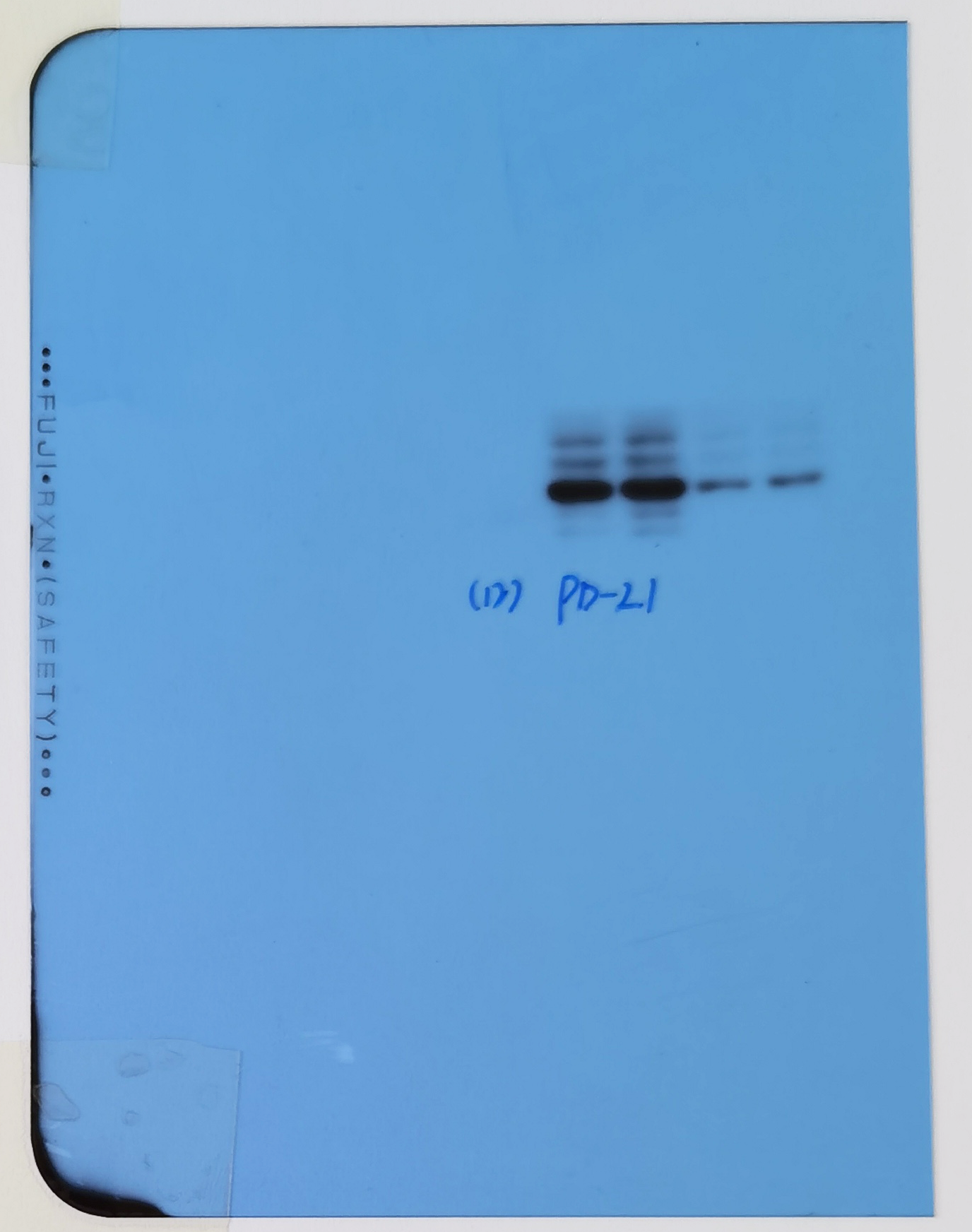

Supplement: Supplementary file 1 — Additional file 1. [file 12957_2023_2969_MOESM1_ESM.zip › PD-L1 (fig5A right).tif]

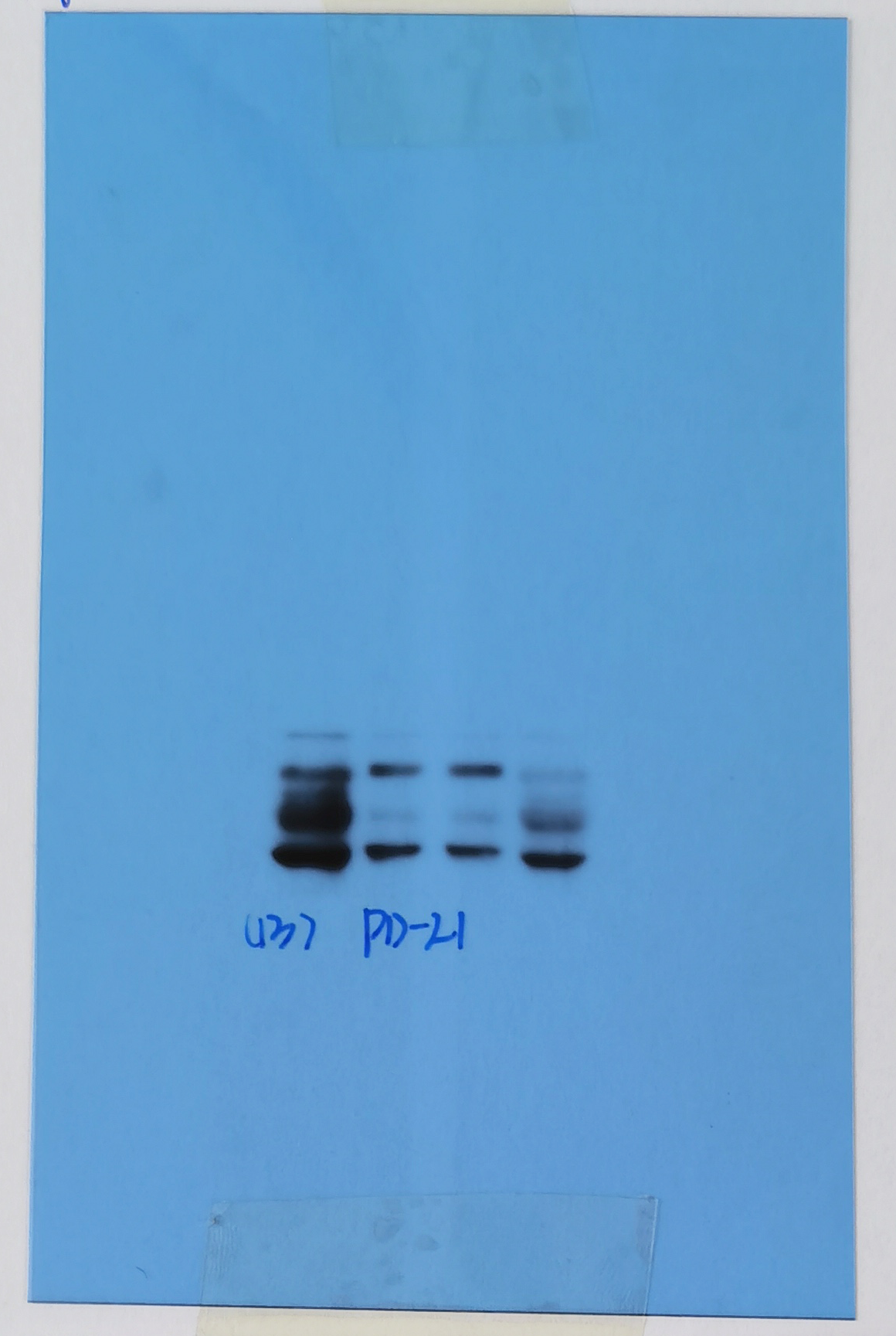

Supplement: Supplementary file 1 — Additional file 1. [file 12957_2023_2969_MOESM1_ESM.zip › PD-L1 (fig6A).tif]
